# Tissue-type plasminogen activator controls neuronal death by raising surface dynamics of extrasynaptic NMDA receptors

**DOI:** 10.1038/cddis.2016.279

**Published:** 2016-11-10

**Authors:** Flavie Lesept, Arnaud Chevilley, Julie Jezequel, Laurent Ladépêche, Richard Macrez, Margaux Aimable, Sophie Lenoir, Thomas Bertrand, Laëtitia Rubrecht, Pascale Galea, Laurent Lebouvier, Karl-Uwe Petersen, Yannick Hommet, Eric Maubert, Carine Ali, Laurent Groc, Denis Vivien

**Affiliations:** 1 Inserm, Inserm UMR-S U919, Serine Proteases and Pathophysiology of the Neurovascular Unit, Université Caen-Normandie, GIP Cyceron, Caen, France; 2 Interdisciplinary Institute for Neuroscience, Université de Bordeaux UMR 5297, Bordeaux, France; 3 CNRS IINS UMR 5297, Bordeaux, France; 4 SysDiag CNRS/Bio-Rad, UMR3145, Montpellier, France; 5 PAION Deutschland GmbH, Aachen, Germany

## Abstract

*N*-methyl-d-aspartate receptors (NMDARs) are ion channels whose synaptic *versus* extrasynaptic localization critically influences their functions. This distribution of NMDARs is highly dependent on their lateral diffusion at the cell membrane. Each obligatory subunit of NMDARs (GluN1 and GluN2) contains two extracellular clamshell-like domains with an agonist-binding domain and a distal N-terminal domain (NTD). To date, the roles and dynamics of the NTD of the GluN1 subunit in NMDAR allosteric signaling remain poorly understood. Using single nanoparticle tracking in mouse neurons, we demonstrate that the extracellular neuronal protease tissue-type plasminogen activator (tPA), well known to have a role in the synaptic plasticity and neuronal survival, leads to a selective increase of the surface dynamics and subsequent diffusion of extrasynaptic NMDARs. This process explains the previously reported ability of tPA to promote NMDAR-mediated calcium influx. In parallel, we developed a monoclonal antibody capable of specifically blocking the interaction of tPA with the NTD of the GluN1 subunit of NMDAR. Using this original approach, we demonstrate that the tPA binds the NTD of the GluN1 subunit at a lysine in position 178. Accordingly, when applied to mouse neurons, our selected antibody (named Glunomab) leads to a selective reduction of the tPA-mediated surface dynamics of extrasynaptic NMDARs, subsequent signaling and neurotoxicity, both *in vitro* and *in vivo*. Altogether, we demonstrate that the tPA is a ligand of the NTD of the obligatory GluN1 subunit of NMDAR acting as a modulator of their dynamic distribution at the neuronal surface and subsequent signaling.

*N*-methyl-d-aspartate receptors (NMDARs) are tetrameric assemblies of dimers of GluN1 and GluN2 (GluN2A-D) subunits (possibly GluN3A-B). Their extracellular region forms a massive protrusion composed of eight clamshell-like domains arranged in two layers: a distal N-terminal domain (NTD) layer and a layer of four agonist-binding domains (ABDs) directly connected to the transmembrane domain (TMD).^1^ Subunit composition and synaptic *versus* extrasynaptic localization critically influence NMDAR functions.^2^ Synaptic *versus* extrasynaptic distribution of NMDARs is highly dependent on their lateral diffusion at the cell membrane.^3, 4, 5^ It is interesting to note that this diffusion can be modulated by extracellular factors such as matrix metalloproteases or co-agonists.^6, 7, 8^ In cortical and hippocampal areas, NMDARs are principally composed of GluN1 subunits that are associated with GluN2A and GluN2B.^9^ In contrast to GluN2 subunit NTDs,^10^ less is known about the obligatory role and dynamics of the NTD of the GluN1 subunit (GluN1 NTD) in NMDAR allosteric signaling. A recent work by Zhu *et al.*^10^ show that GluN1 NTD is highly mobile and actively participates in defining the gating and pharmacological profile of NMDARs. These data suggested that any ligand that binds GluN1 NTD may stabilize its opened or closed conformations and thus act as a positive, or negative, allosteric modulator of NMDAR.

Tissue-type plasminogen activator (tPA), a serine protease of 69 kDa, is expressed in most organs, including the brain and the spinal cord.^11, 12, 13, 14^ It consists of five different functional domains through which it interacts with various substrates, binding proteins and receptors.^15, 16^ In the central nervous system (CNS), tPA can be synthesized and released by virtually all cell types. This neuromodulator displays an array of important functions, which are involved in synaptic plasticity,^17^ learning and memory processes,^18, 19^ anxiety ^20^ and neuronal survival or death.^14, 21, 22, 23^ Although previous studies demonstrated that tPA was a modulator of NMDARs signaling through a possible interaction with the GluN1 NTD,^22, 24^ the exact molecular mechanism of this function remains under debate.^10^

In this study, we postulated that tPA could alter NMDAR-evoked signaling and subsequent neurotoxicity through modifications of their surface dynamics and distribution. Thus, using nanoparticle tracking and antibody-based screening in living neurons, we demonstrate that the neuronal extracellular serine protease tPA can selectively increase neuronal extrasynaptic NMDARs surface diffusion. This selective diffusion of NMDAR is the consequence of a direct interaction of tPA with a functionally critical single amino acid (lysine 178) within the GluN1 NTD. By this mechanism, tPA promotes NMDAR-dependent calcium influx and excitotoxic neuronal death both *in vitro* and *in vivo*.

## Results

### tPA selectively increases extrasynaptic NMDAR surface dynamics, clustering and subsequent signaling

**Figure 1:**
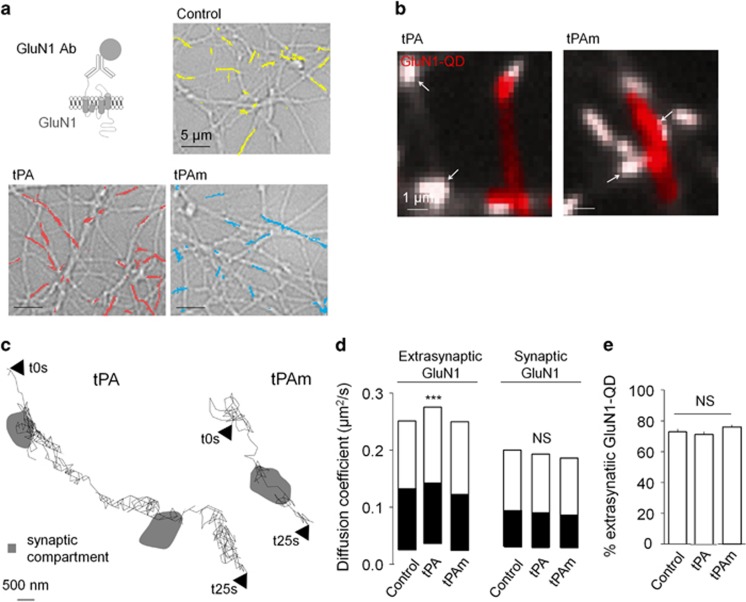
tPA selectively increases extrasynaptic GluN1-NMDAR surface diffusion. (**a**) Schematic representation of the surface labeling of a GluN1 subunit using a single anti-GluN1 antibody QD complex. Representative GluN1-QD (GluN1 QD) trajectories on cultured hippocampal neurons (11–12 DIV) incubated 45 min with buffer (yellow), WT tPA (300 nM, red) or a non-proteolytic tPA (tPAm, 300 nM, cyan). (**b**) Representative trajectories of surface GluN1-QD (red lines, 500 frames, 50-ms acquisition) in the vicinity and within synapses (white arrows). Synaptic trajectories are defined by their colocalization with synaptic labeling (Mitotracker, white), trajectories outside synapses being considered as extrasynaptic. (**c**) Representative trajectories of GluN1-NMDAR tracked with a control GluN1 NTD antibody (control Ab) in the presence of tPA or tPAm (300 nM both). (**d**) Instantaneous diffusion coefficient distributions (median 25–75% IQR,) of extrasynaptic (control *n*=3882 trajectories; tPAm *n*=3195; tPA *n*=3701; *N*=3 independent experiments; ***P<0.001, Kruskal–Wallis test followed by Dunn's multiple comparison test) *versus* synaptic GluN1-QD (control *n*=4411 trajectories; tPAm *n*=2818; tPA *n*=4283). Note that GluN1 surface diffusion is specifically increased in the extrasynaptic compartment after tPA incubation. (**e**) Fraction of extrasynaptic GluN1-QD is unchanged between the different incubations (control 73%, *n*=18; tPAm 76%, *n*=20; tPA 71%, *n*=17; NS, not significant, one-way ANOVA; mean±S.E.M.)

In living hippocampal culture, single nanoparticle tracking was used to image surface NMDARs and their response to exogenously applied tPA (see Materials and methods section; Figure 1). NMDAR surface diffusion was recorded in cultured neurons that were incubated with single nanoparticle complexes containing a quantum dot (QD) associated with polyclonal antibodies raised against GluN1 NTD (Figure 1a). Surface diffusion of GluN1-NMDAR was recorded under control conditions and in the presence of either a wild-type (WT) tPA (tPA, 300 nM; Figure 1a) or a non-proteolytic tPA (tPAm, 300 nM). Figures 1a to c illustrate, at different magnifications, the distinct diffusion patterns of representative trajectories of surface NMDAR following exposure to either WT tPA or tPAm. We then compared the diffusion NMDAR to discriminate potential compartment-specific effect of tPA. As shown previously, synaptic NMDAR are less diffusive than their extrasynaptic counterparts.^3, 25^ Although the diffusion of synaptic receptors remained unchanged, exposure to tPA increased the diffusion of extrasynaptic receptors (***P<0.001) (Figures 1c and d). In contrast to its WT form, inactive tPAm failed to influence the dynamics of NMDARs. It should be noted that these acute applications of tPA did not modify the percentage of detected extrasynaptic GluN1-NMDAR. This indicates a stable pool of membrane NMDAR within this time frame (Figure 1e). Altogether, these data provide the first direct evidence that tPA can increase extrasynaptic,but not synaptic, GluN1-NMDAR surface dynamics in living neurons.

**Figure 2:**
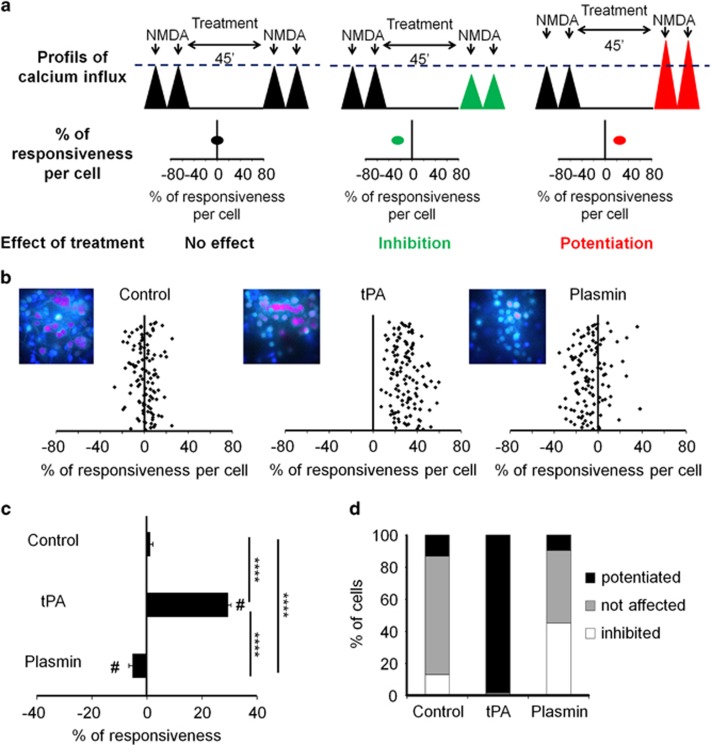
tPA-induced potentiation of NMDAR-mediated neuronal calcium influx is independent of plasmin. (**a**) Schematic representation of calcium video imaging on primary cultures of cortical neurons (12–14 DIV). The neurons were stimulated twice with the NMDA, then incubated with the treatment during 45 min prior a second run of NMDA stimulations. Each neuron responsive to NMDA is thus its own control. By comparison between the two runs of NMDA stimulations (% of responsiveness per cell), we visualized the effect of treatment on NMDA-induced calcium influx estimated the % of responsiveness per cell with either no effect of the treatment, an inhibitory effect or a potentiating effect. Each dot represents one individual neuron, with data collected from a minimum of three independent experiments. (**b**) After control NMDA stimulations used as baseline, neurons were incubated for 45 min with either plasmin buffer (control, *n*=107 cells), tPA (300 nM, *n*=123 cells) or plasmin (100 *μ*g/ml; *n*=115 cells). Each dot represents one cell. (**c**) Percentage of stimulation or inhibition after incubation were calculated for each individual cell and reported as the percentages of responsiveness for each group (mean±S.E.M.; *N*=3 independent experiments; ****P<0.0001 Kruskal–Wallis and Mann–Whitney tests for group comparison; ^#^*P*<0.0001 Wilcoxon signed-rank test for the comparison pre- and post-incubation responses). (**d**) Represents the percentages of potentiated, not affected and inhibited cells

**Figure 3:**
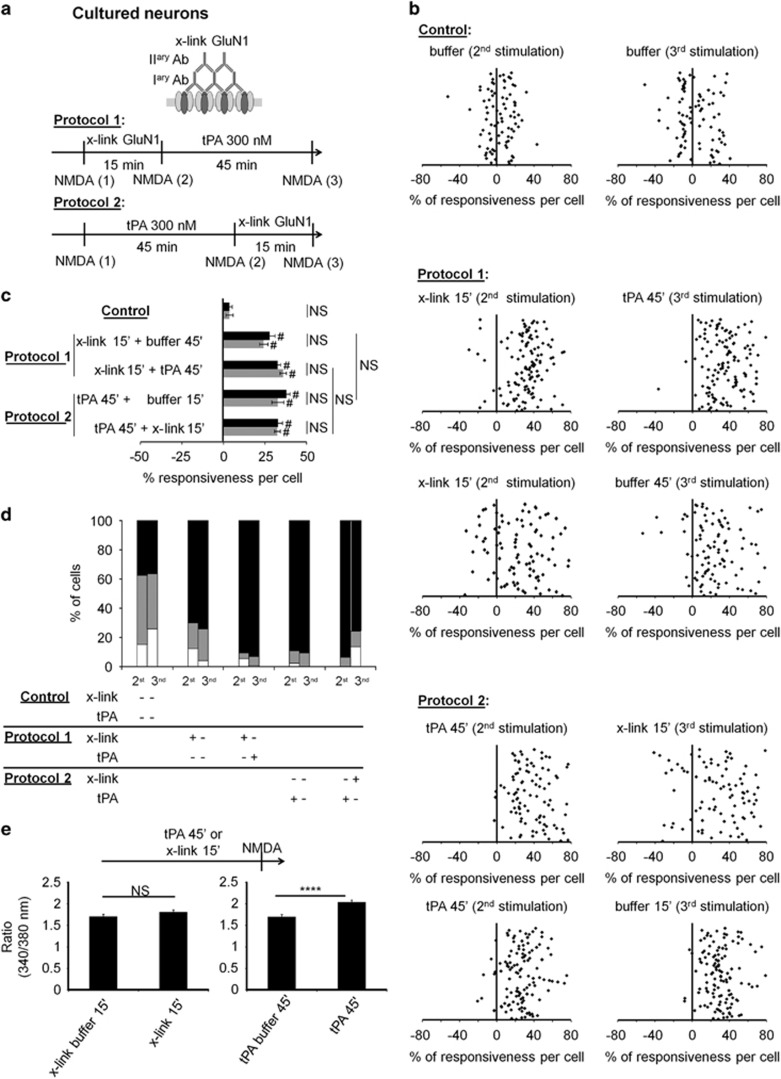
tPA mimicks x-link-induced NMDARs clustering. (**a**) Schematic representation of the x-link procedure performed on cultured cortical neurons and used to induce an artificial clustering of GluN1-NMDAR,^26, 27, 28^ as well as the detailed sequence of treatments. (**b**) NMDA-induced calcium influx measured after the following treatments: buffer alone (*n*=85 cells), x-link 15' then tPA 45' (protocol 1, *n*=128 cells) or tPA buffer 45'(*n*=97 cells) and tPA 45' then x-link 15' (protocol 2, *n*=95 cells) or x-link buffer 15' (*n*=114 cells). Each dot represents one cell. (**c**) Percentage of stimulation or inhibition after incubation were calculated for each individual cell and reported as percentages of responsiveness for each group. (mean±S.E.M.; *N*=3 independent experiments; NS, not significant; ****P<0.0001 Kruskal–Wallis test followed by Mann–Whitney test for group comparison; ^#^*P*<0.0001 Wilcoxon signed-rank test for the comparison pre- and post-incubation responses). (**d**) Percentages of potentiated, not affected and inhibited cells. (**e**) Comparison of the amplitudes of calcium influx after tPA 45' (*n*=87 cells) or x-link 15' (*n*=77 cells) treatments and their controls (tPA buffer 45', *n*=68 cells; x-link buffer 15', *n*=92 cells) without a pre-treatment with NMDA (as shown on the diagram). Effects of tPA 45' or x-link 15' are not dependent of the pre-incubation NMDA stimulation. In contrast to tPA 45' condition, the x-link does not lead a augmentation of calcium influx amplitude. (mean±S.E.M.; *N*=3 independent experiments; ****P<0.0001 Mann–Whitney test for group comparison)

Next, we investigated the influence of tPA on consecutive NMDA-evoked calcium influx (Figure 2). The neurons were stimulated twice with the NMDA, then incubated with the treatment during 45 min prior a second run of NMDA stimulations. Each neuron responsive to NMDA is thus its own control. By comparison between the two runs of NMDA stimulations (% of responsiveness per cell), we visualized the effect of treatment on NMDA-induced calcium influx estimated by the % of responsiveness per cell with either no effect of the treatment, an inhibitory effect or a potentiating effect. Each dot represents one individual neuron, with data collected from a minimum of three independent experiments (Figure 2a). tPA treatment increased NMDA-induced calcium influx (300 nM; 92% of neurons potentiated, +28% stimulation in mean value; ****P<0.0001 when compared with control neurons; Figures 2b–d). This effect is independent of the plasmin because plasmin did not elicit such a potentiation of NMDA-evoked calcium influx (Figure 2). To directly address whether this functional effect of tPA on NMDA-induced calcium influx response requires, or could be mimicked by, surface diffusion of NMDAR. We artificially reduced NMDAR surface dynamics and created NMDAR clusters using a cross-link protocol (x-link)^26, 27, 28^ (Figure 3a). It should be noted that x-link alone, without NMDA application, does not alter the amplitude of calcium influx, as previously reported^28, 29^ (Figure 3e). Remarkably, after a first challenge with NMDA, x-link led to an increase of NMDA-induced calcium influx (90% of neurons potentiated, +33% stimulation in mean value; control *n*=85 cells and x-link *n*=128 cells; ****P<0.0001 when compared with control cells), indicating that NMDA-induced calcium influx response requires dynamic diffusion of surface NMDAR. The same effect was observed following tPA treatment (94% of cells potentiated, +38% stimulation in mean value; protocol 2, *n*=95 cells; ****P<0.0001 when compared with control cells; Figures 3c and d). Importantly, no additional effect on calcium response was observed when adding tPA after performing x-link (NS, not significant, protocol 1), or when performing x-link after adding tPA (NS; protocol 2; Figures 3b–d). The same results were obtained when x-link was induced by Glunomab (Supplementary Figure 1) instead of polyclonal antibodies against GluN1 NTD subunit (residues 385–399, Figure 3). Altogether, these data demonstrate that NMDA-induced calcium influx requires a fast dynamic diffusion of NMDAR, allowing clustering, as shown by x-link experiments. For extrasynaptic NMDARs, this process is promoted by tPA.

### Promotion of NMDARs signaling is caused by tPA interaction with GluN1: critical role of lysine 178 (GluN1 NTD^Lys178^)

**Figure 4:**
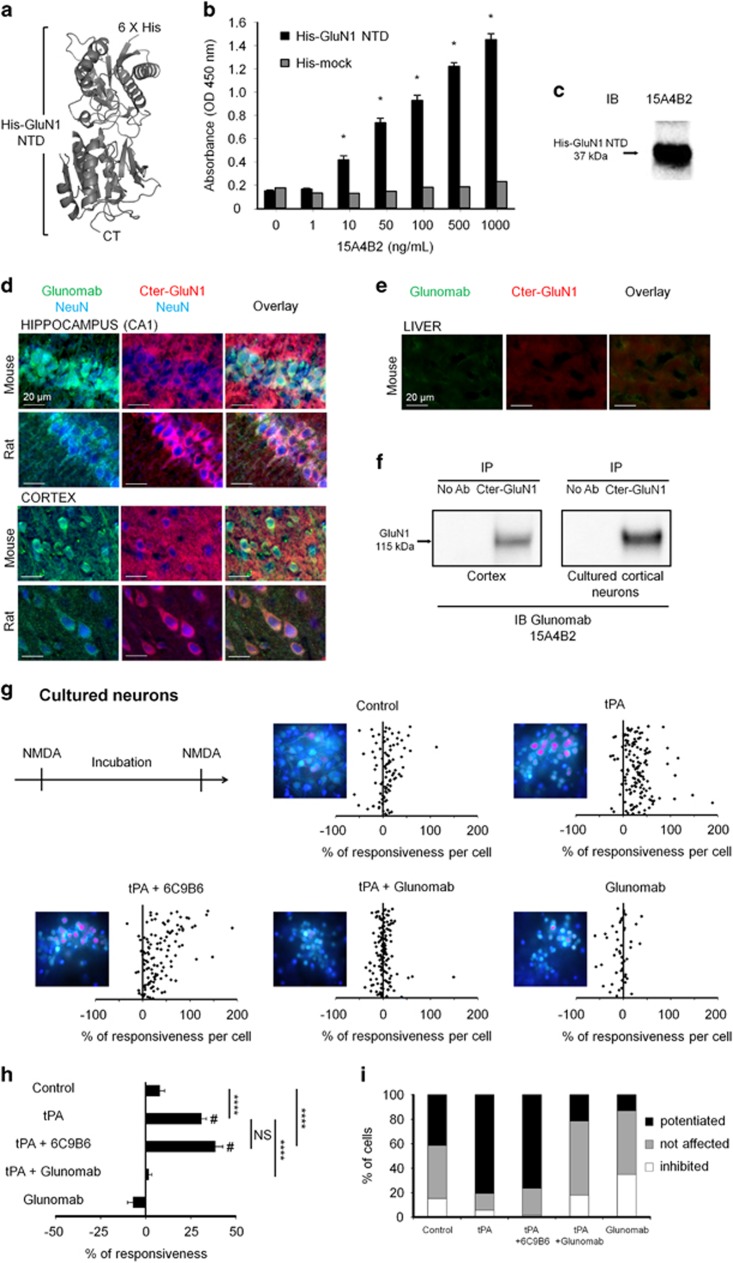
Production and characterization of Glunomab (clone 15A4B2), a monoclonal antibody raised against GluN1 NTD. (**a**) Structure of the bilobate amino-terminal domain of GluN1 (GluN1 NTD, PDB ID: 3Q41).^50^ The GluN1 NTD was used as antigen for immunization assays in mice. (**b**) Example of an indirect ELISA assay performed with conditioned media harvested from Glunomab (clone 15A4B2), using a His-tagged mock protein as a control or the His-GluN1 NTD as the specific antigen (1–1000 ng/ml of antibody, *N*=4 independent experiments; *P<0.05 compared between one dose and previous dose; Kruskal–Wallis and Mann–Whitney tests). (**c**) Immunoblotting performed after SDS-PAGE resolution of recombinant His-GluN1 NTD (20 *μ*g loaded per lane) and immunoglobulin Glunomab (clone 15A4B2) as the primary antibody (*N*=2 experiments). (**d**) Immunostaining performed from hippocampal (CA1) and cortical tissue sections (mice and rats) using either Glunomab (clone 15A4B2) (1 : 400, green) or an antibody against the C-terminal GluN1 subunit (Cter-GluN1, 1 : 400, red) or NeuN (1 : 250, blue). (**e**) Control immunostainings performed from liver tissue sections (mice) using either Glunomab (clone 15A4B2) (1 : 400, green) or an antibody against the C-terminal GluN1 subunit (Cter-GluN1, 1 : 400, red). (**f**) Immunoprecipitation (IP) assays from mouse cortices or primary cultures of cortical neurons using Cter-GluN1 subunit antibody followed by immunoblotting (IB) using Glunomab (clone 15A4B2) (representative data of three independent samples). (**g**) Calcium video imaging was performed on primary cultures of cortical neurons (12–14 DIV, see Materials and Methods section). After two successive control NMDA stimulations, neurons were incubated for 45 min with buffer (control, *n*=98 cells), Glunomab alone (10 *μ*g/ml, *n*=46 cells), tPA alone (300 nM, *n*=139 cells) or with immunoglobulins corresponding to Glunomab (clone 15A4B2) (10 *μ*g/ml, *n*=139 cells) or the clone 6C9B6 (10 *μ*g/ml, *n*=119 cells). A second set of NMDA stimulations was then performed. Each dot represents one cell. Each graph is accompanied by an image during NMDA stimulation after treatment. (**h**) Percentage of stimulation or inhibition after incubation were calculated for each individual cell and reported as percentages of responsiveness for each group (mean±S.E.M.; *N*=3 independent experiments; NS, not significant; ****P<0.0001; Kruskal–Wallis and Mann–Whitney tests for group comparison; ^#^*P*<0.0001 Wilcoxon signed-rank test for the comparison pre- and post-incubation responses). (**i**) Percentages of potentiated, not affected and inhibited cells

To identify the putative binding site of tPA on GluN1 NTD,^22, 30, 31, 32^ we first screened a series of monoclonal antibodies we have generated using recombinant GluN1 NTD as antigen (residues 19–371; Figure 4a). Positive antibodies on ELISA (Figure 4b and Supplementary Figure 2) were further selected by immunoblottings raised against recombinant GluN1 NTD (Figure 4c and Supplementary Figure 2). Then, histochemistry performed with selected antibodies (e.g., clone 15A4B2 also named Glunomab) revealed positive stainings in cortical and hippocampal neurons (mouse and rat). Glunomab-positive staining colocalizes with a parallel immunostaining performed against the C-terminal end of GluN1 (Cter-GluN1) both in cortical and hippocampal but not in non-neuronal tissue sections (Figures 4d and e). As Glunomab is not optimum for direct immunoblotting, we tested its specificity after we have performed a pull down of the GluN1 subunit by using an antibody raised against the Cter-GluN1 (Figures 4e–g. clone 15A4B2, Glunomab). These experiments were performed from whole cortex and primary cultures of cortical neurons protein extracts. We then evaluated the blocking activity of each positive clone on tPA-driven potentiation of NMDAR signaling using calcium video-imaging assays performed on primary cultures of cortical neurons (Figures 4g–i). NMDA exposure led to reproducible waves of calcium influx that were potentiated by 31% in the presence of tPA (300 nM; mean value of potentiation with 81% of cells potentiated; ****P<0.0001; control, *n*=92 cells and tPA, *n*=139). Antibodies from clone 6C9B6 failed to influence tPA-induced potentiation of NMDA-induced calcium influx (+38% of potentiation with 78% of cells potentiated; *n*=119 cells; ^#^*P*<0.0001 compared with pre-incubation responses; NS, not significant compared with tPA alone). However, Glunomab completely abolished the tPA-dependent potentiation of NMDA-induced calcium influx (****P<0.0001 when compared with tPA alone, *n*=139 cells). Of note, Glunomab did not affect basal NMDAR signaling (*n*=46 cells).

Then, an epitope mapping of Glunomab was performed to refine the binding site of tPA on the GluN1 NTD. Immunoblottings for 141 overlapping pentadecapeptides frame shifted by three residues covering the entire amino-acid sequence of GluN1 NTD (residues 19–371) were revealed with Glunomab (Supplementary Figure 3a). Once reactive peptides were identified, a corresponding alanine scanning (Alascan) was performed to identify key residues contributing to the epitope of Glunomab (Supplementary Figure 3b). These studies revealed that Glunomab binds to the ^172^EGRAAQKRLETLLEE^186^ sequence of the rat GluN1 NTD, a sequence 100% conserved between species from mouse to human (Supplementary Figures 3c and d). Alascan also revealed ^176^AQKRL^180^ as the minimal epitope and both arginine 174 (R174) and glutamic acid 185 (E185) as putative stabilizing amino acids. Positioning of the minimal epitope (^176^AQKRL^180^) in the structure of heterotetrameric NMDAR reveals that this epitope is accessible to extracellular molecules (Supplementary Figure 3e).

**Figure 5:**
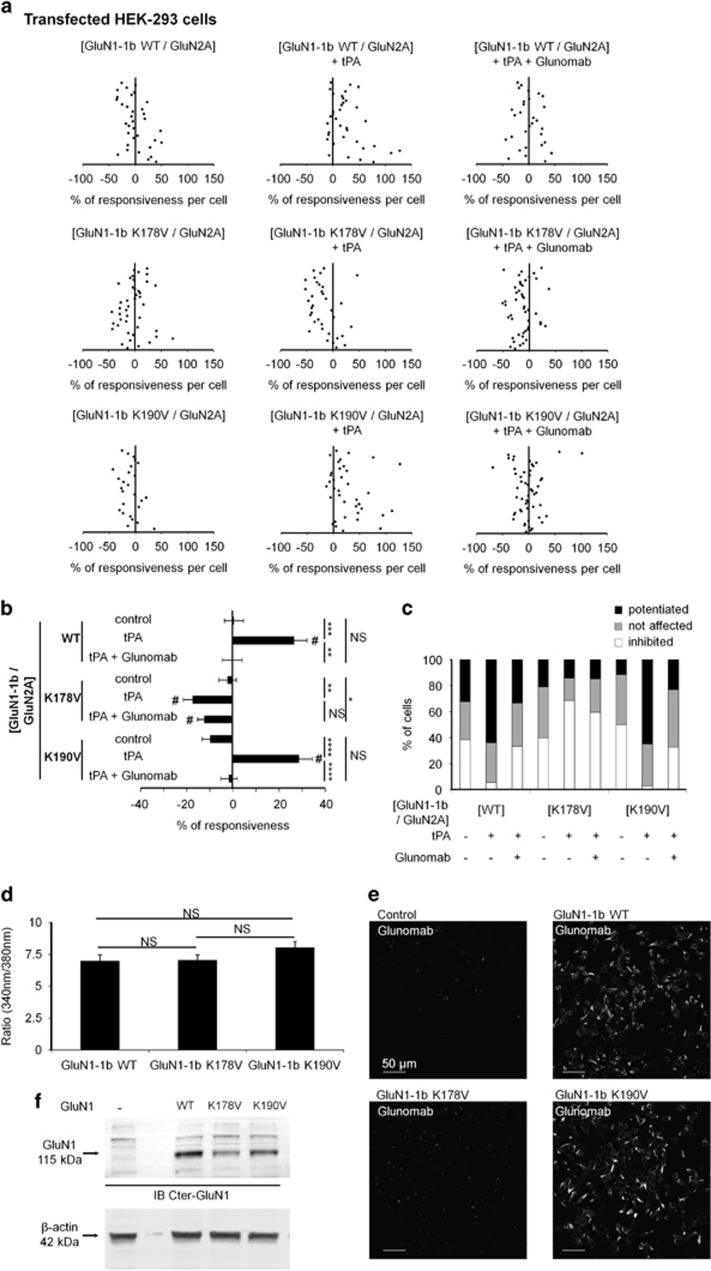
tPA-induced potentiation of NMDAR signaling involves the lysine 178 of the GluN1 NTD (GluN1 NTD^Lys178^). (**a**) Calcium video imaging performed on HEK-293 cells transiently transfected with either GluN1-1b WT, K178V mutated GluN1-1b or K190V mutated GluN1-1b in combination with WT GluN2A. After control NMDA stimulations (used as baseline), transfected cells with GluN1-1b WT and GluN2A were incubated for 20 min with either buffer (control, *n*=34 cells), tPA (300 nM, *n*=36 cells) and/or Glunomab (10 *μ*g/ml, *n*=30 cells), before a second set of NMDA stimulations. The same set of NMDA stimulations were applied on cells transfected with mutated GluN1-1b K178V or mutated GluN1-1b K190V incubated with either buffer (*n*=43 cells or *n*=26, respectively) or tPA (300 nM, *n*=35 cells or *n*=37, respectively) or tPA with Glunomab (*n*=47 cells and *n*=52 cells, respectively). Each dot represents one cell. (**b**) Percentage of stimulation or inhibition after incubation were calculated for each individual cell and reported as the percentages of responsiveness for each group (mean±S.E.M.; *N*=3 independent experiments; *P<0.05; **P<0.01; ***P<0.001; ****P<0.0001 Kruskal–Wallis and Mann–Whitney tests for group comparison; ^#^*P*<0.01 Wilcoxon signed-rank test for the comparison pre- and post-incubation responses). (**c**) Percentages of potentiated, not affected and inhibited cells. (**d**) Calcium video imaging performed on HEK-293 cells transiently transfected with either GluN1-1b WT, K178V mutated GluN1-1b or K190V mutated GluN1-1b in combination with WT GluN2A. Before an incubation with different treatment, two first NMDA stimulations (100 *μ*M) were performed on transfected HEK-293 cells with GluN1-1b WT (or K178V or K190V) and GluN2A. The K178V and K190V point mutations within GluN1 do not influence the basal activity of NMDAR in the absence of tPA. (mean±S.E.M.; *N*=3 independent experiments; NS, not significant; Kruskal–Wallis and Mann–Whitney tests for group comparison). (**e**) Representative images of immunolabelings revealed with Glunomab (as primary antibody, 1 : 800) performed on HEK-293 cells transiently transfected with either GluN1-1b WT, mutated GluN1-1b K178V or mutated GluN1-1b K190V in combination with WT GluN2A (*N*=3 independent experiments). (**f**) Immunoblotting raised against GluN1 (Cter-GluN1, 1 : 250) performed from protein extracts of HEK-293 cells transiently transfected with either GluN1-1b WT, K178V mutated GluN1-1b or K190V mutated GluN1-1b in combination with WT GluN2A and a loading control using β-actin antibody (20 *μ*g loaded per lane, *N*=1 experiment)

We demonstrated previously that the lysine-binding site (LBS) of the kringle 2 domain of tPA was important to promote NMDAR signaling.^33, 34^ Thus, we postulated that the lysine 178 (K^178^) in the minimal epitope of Glunomab could be critical for the binding of tPA on GluN1 NTD. To address this question, NMDA-induced calcium influx was recorded in HEK-293 cells transfected with WT GluN2A in combination with either WT GluN1-1b (GluN1-1b WT) or GluN1-1b containing a point mutation of the lysine in position 178 into valine (K178V) or GluN1-1b containing a point mutation of lysine in position 190 into valine as control (K190V) (Figures 5a–c). As observed in cultured cortical neurons, tPA enhanced NMDA-induced calcium influx (+27% of potentiation with 62% of cells potentiated; ***P<0.001 compared with control; control, *n*=34 cells; tPA, *n*=36) and Glunomab prevented tPA-induced potentiation of NMDAR signaling (**P<0.01 when compared with tPA alone; *n*=30 cells). K178V mutated NMDAR were still responsive to NMDA stimulation but failed to respond to tPA (*n*=35 cells). The control point mutation K190V did not modify NMDAR responsiveness to tPA incubation (*n*=37 cells; NS compared with tPA alone), neither the blocking activity of Glunomab (****P<0.0001 compared with GluN1-1b K190V in tPA condition; *n*=47 cells). These mutations do not influence the basal activity of NMDAR in the absence of tPA (Figure 5d). Accordingly, binding of Glunomab was reduced on HEK-293 cells transfected with GluN1-1b K178V compared with cells expressing NMDARs containing either GluN1-1b WT or GluN1-1b K190V (Figures 5e and f). Taken together, these data demonstrate that lysine 178 within the GluN1 NTD is of decisive importance in effecting tPA-induced potentiation of NMDAR signaling and, equally important, these findings revealed Glunomab as a selective antagonist of tPAs action on NMDAR.

**Figure 6:**
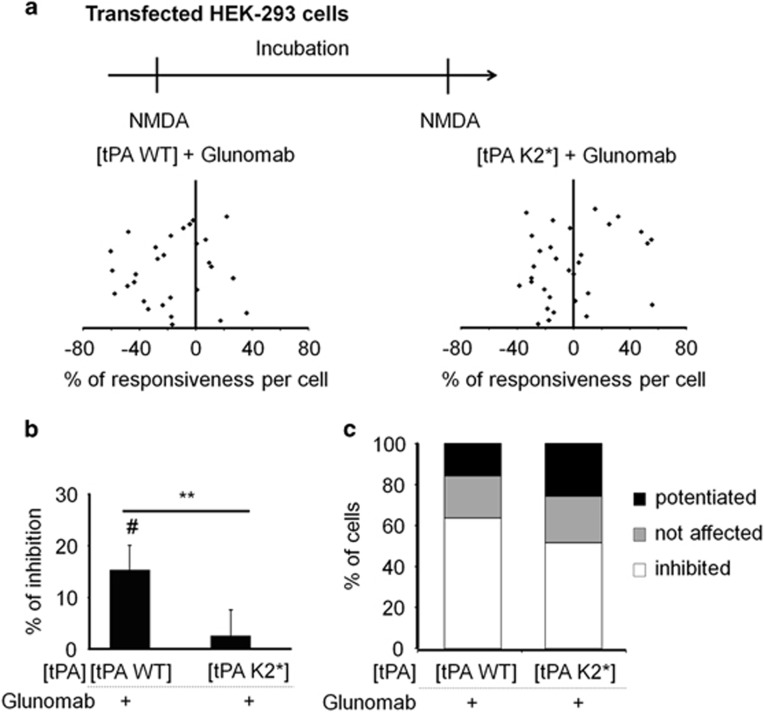
tPA-induced potentiation of NMDAR signaling is dependent of the LBS of the tPA kringle 2 domain (kringle 2-tPA^LBS^). (**a**) Calcium video imaging performed on HEK-293 cells transiently transfected with GluN1-1b WT and GluN2A in combination with tPA WT or a tPA containing a point mutation within its LBS-containing kringle 2 domain (W254, tPA K2*).^35^ After control NMDA stimulations (used as baseline), transfected HEK-293 cells with either GluN1-1b/GluN2A/tPA WT (*n*=44 cells) or GluN1-1b/GluN2A/tPA K2* (*n*=31 cells) were incubated for 20 min with Glunomab (10 *μ*g/ml), before a second set of NMDA stimulations. (**b**) Percentages of inhibition after incubation with Glunomab were calculated for each individual cell and reported as the percentages of inhibition for each group (mean±S.E.M.; *N*=3 independent experiments; **P<0.01 Kruskal–Wallis and Mann–Whitney tests for group comparison; ^#^*P*<0.01 Wilcoxon signed-rank test for the comparison pre- and post-incubation responses). (**c**) Percentages of potentiated, not affected and inhibited cells

To ascertain whether the LBS contained in the kringle 2 domain of tPA (K2) was indeed the tPA moiety that interacts with the lysine 178 of the GluN1 NTD, we used a previously characterized mutant of tPA containing an inactive LBS (W254R), hereafter termed tPA K2*.^33^ NMDA-induced calcium influx was recorded in HEK-293 cells transfected with rat cDNAs encoding both GluN1-1b WT and GluN2A in combination with WT rat tPA (tPA WT) or tPA K2* (Figure 6a). Although Glunomab reduced NMDAR signaling recorded in the presence of tPA WT (–18% of potentiation with 62% of cells inhibited; ^#^*P*<0.01; *n*=29 cells), it failed to reduce NMDA-induced calcium influx recorded in the presence of tPA K2* (**P<0.01 compared with tPA WT; *n*=19 cells; Figures 6b and c). These data show that Glunomab is specific of the tPA/NMDAR interaction directly involving the LBS of tPA and the lysine in position 178 within GluN1 NTD.

### Preventing tPA/GluN1 NTD interaction reduces extrasynaptic NMDAR diffusion, tPA-promoted NMDAR signaling and subsequent neurotoxicity

**Figure 7:**
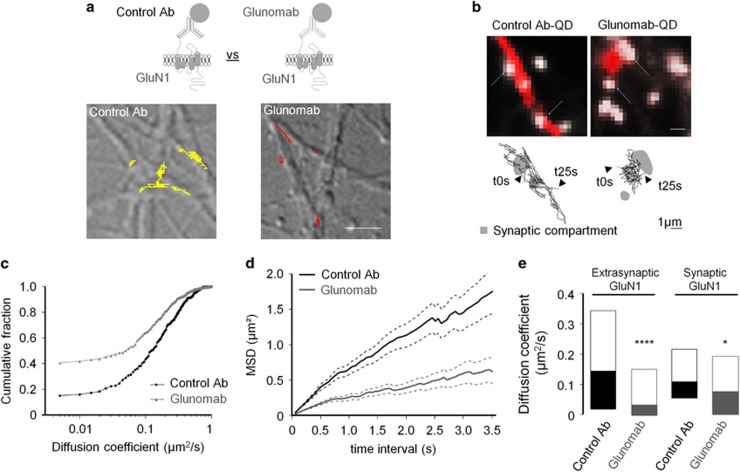
Blockage of the ability of tPA to bind GluN1 NTD alters extrasynaptic GluN1-NMDARs surface diffusion and increases their confined behavior. (**a**) Up: GluN1-NMDAR tracked using a single anti-GluN1 antibody QD complex obtained either by coupling a control GluN1 NTD subunit antibody (control Ab, Alomone Labs, 1 : 200) or the Glunomab antibody (1 : 200) to QDs. Down: representative GluN1-QD (GluN1 QD) trajectories on cultured hippocampal neurons (11–12 DIV) with control Ab (yellow) or Glunomab (red). (**b**) Representative trajectories of surface GluN1-QD (black lines, 500 frames, 50-ms acquisition) in the vicinity and within synapses (white arrows). Synaptic trajectories are defined by their colocalization with synaptic labeling (Mitotracker, white), trajectories outside synapses being considered as extrasynaptic. Note that the diffusion of NMDAR targeted by Glunomab is reduced outside synapses. Scale bar=1 *μ*m. (**c**) Cumulative distribution of the instantaneous diffusion coefficient of NMDARs targeted by the control Ab or Glunomab. The population of NMDAR targeted by Glunomab shows a reduced diffusion speed and a higher proportion of immobile receptors compared with the one tracked with the control Ab (immobile fraction Control Ab=15% Glunomab=41%). (**d**) Plot of the MSD *versus* time of total GluN1 tracked with control Ab (*n*=273 trajectories) or Glunomab (*n*=129). The red curve (Glunomab) tends toward a negative curvature, characteristic of a confined behavior. (**e**) Instantaneous diffusion coefficient distributions (median 25–75% IQR) of extrasynaptic (control Ab=0.1489 *μ*m^2^/s IQR=0.0228–0.3476 *μ*m^2^/s, *n*=148 trajectories; Glunomab=0.0318 *μ*m^2^/s IQR=0.00008–0.1532 *μ*m^2^/s, *n*=261) *versus* synaptic GluN1-QD (control Ab=0.1132* μ*m^2^/s IQR=0.0511–0.2148 *μ*m^2^/s, *n*=170 trajectories; Glunomab=0.0764 *μ*m^2^/s IQR=0.0014–0.1909 *μ*m^2^/s *n*=146; *P<0.05, ****P<0.001; Kruskal–Wallis and Dunn's multiple comparison test)

NMDAR surface diffusion was then recorded on cultured hippocampal neurons incubated with single nanoparticle complexes containing a QD associated with either an antibody raised against GluN1 NTD subunit or Glunomab. These experiments were designed to assess the effects of endogenous tPA on the dynamics of NMDAR at the neuronal surface. Figures 7a and b illustrate the distinct diffusion patterns from representative trajectories of surface NMDAR, labeled with control antibodies (control Ab) or Glunomab. The epitopes of the control Ab and of Glunomab are different corresponding, respectively, to the amino-acid residues 385–399 and 176–180 of the GluN1 subunit. However, both control Ab and Glunomab can bind GluN1 NTD. In consequence, the striking reduction in receptor surface diffusion by Glunomab compared with the control antibody can be only explained by the specificity of Glunomab, that is, its capacity to prevent the interaction of tPA with the GluN1 subunit of the NMDAR. Indeed, the fraction of immobile receptors increased more than twofold (Figure 7c). When the curve of the average square displacement (MSD) over time was calculated for the two conditions, the Glunomab curve was shifted to the right, indicating a stronger confined behavior of NMDAR in this condition (Figure 7d). We then recorded and specifically compared the diffusion of synaptic and extrasynaptic NMDAR to discriminate potential compartment-specific effect of tPA binding site. Although the reduced diffusion observed with Glunomab was marked for extrasynaptic receptors (****P<0.0001; control Ab, *n*=170 trajectories; Glunomab, *n*=146), only a slight reduction was observed for synaptic receptors (*P<0.05; control Ab, *n*=148 trajectories; Glunomab, *n*=261; Figure 7e). Altogether our data demonstrate that endogenous tPA selectively increases the surface dynamics of extrasynaptic NMDAR and subsequent calcium influx.

**Figure 8:**
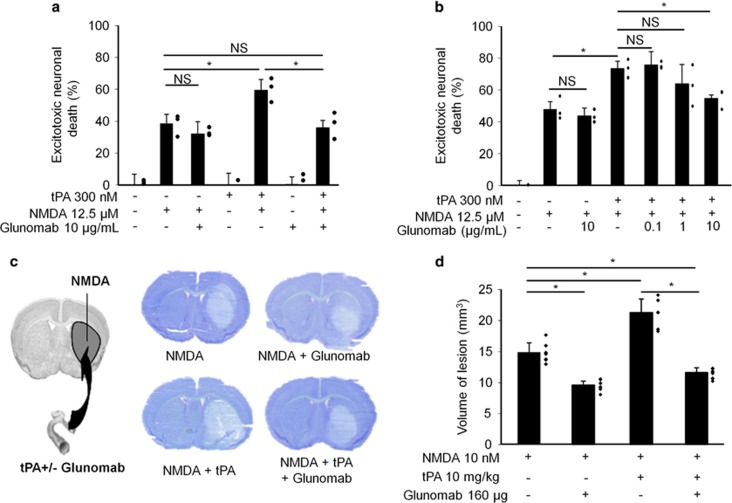
Blockage of the ability of tPA to bind GluN1 NTD interaction prevents the pro-excitotoxic effects of tPA *in vitro* and *in vivo* without alter its anti-apoptotic effect. (**a**) Neuronal death was assessed on primary cultured cortical neurons (12–14 DIV) by measuring LDH release in the bathing media after 24-h exposure to NMDA alone (12.5 *μ*M) or in the presence of tPA (300 nM) and/or Glunomab (10 *μ*g/ml) (mean±S.E.M.; *N*=3 independent experiments, including *n*=9 individual dishes per condition; each spot represents an average value for the three independent experiments; NS, not significant, *P<0.05; Kruskal–Wallis and Mann–Whitney tests). (**b**) Same experiments as in (**a**) were performed in the presence of decreasing concentrations of Glunomab (10, 1, 0.1 *μ*g/ml; mean±S.E.M.; *N*=3 independent experiments, including *n*=9 individual dishes per condition; *P<0.05; Kruskal–Wallis and Mann–Whitney tests; each spot represents an average value for the three independent experiments). (**c**) Left: schematic representation of unilateral striatal injection of NMDA (10 nmoles, 1 *μ*l). Right: illustrative images of excitotoxic lesions in all conditions. (**d**) Quantification of volume of excitotoxic lesions (mean±S.E.M.; *n*=7, 8 or 9 mice per group; *P<0.05 Kruskal–Wallis and Mann–Whitney tests; each spot represents individual lesion volumes).

We then tested whether the blockage of the tPA-dependent potentiation of NMDARs surface dynamics could reduce the pro-neurotoxic action of exogenous tPA in a paradigm of NMDA-mediated neuronal death (Figure 8). NMDA alone (12.5 *μ*M), led to approximately 40% neuronal death 24 h later. Whereas, tPA-promoted NMDA-induced neuronal death (60%, *P<0.05; *n*=3 independent experiments; *n*=9 dishes; Figure 8a), this potentiating effect was prevented in a dose-dependent manner by Glunomab (0.1, 1, 10 *μ*g/ml; *P<0.05; *N*=3 independent experiments; *n*=9 dishes; Figure 8b). *In vivo*, as previously reported,^32^ excitotoxic neuronal death, induced by stereotaxic administration of NMDA (10 nmol) in the mouse striatum, was potentiated by an intravenous injection of tPA (10 mg/kg). This potentiation was not observed if Glunomab (160 *μ*g) was co-administered with tPA (*P<0.05; *n*=7, 8 or 9 per group; Figures 8c and d). We previously showed that tPA promotes the neuronal ERK(½) activation mediating its pro-neurotoxic effects.^35^ Accordingly, we measured the phosphorylated and total ERK(½) levels in primary neuronal cultures subjected to NMDA (50 *μ*M), tPA (300 nM) and Glunomab (10 *μ*g/ml) exposures either alone or in combination (Supplementary Figure 4). After 5 min of treatment, tPA promotes the NMDAR-ERK(½) activation, an effect prevented by the presence of Glunomab (*P<0.05, *N*=5 independent experiments, including a total of *n*=20 individual dishes per condition). These data show that the control of neuronal survival is one of the physio-pathological tPA effects that involve its ability to influence the surface dynamics of extrasynaptic NMDAR.

## Discussion

In this report, we demonstrate that extrasynaptic GluN1-NMDARs surface dynamics and subsequent signaling are increased by the neuronal extracellular serine protease tPA, leading to an enhanced NMDAR signaling and neurotoxicity (Supplementary Figure 5). These effects are the direct consequence of an interaction of tPA with the lysine in position 178 (^176^AQKRL^180^) of the GluN1 NTD of NMDAR. Thus, tPA acts as a modulator of NMDARs distribution at the neuronal surface.

In the CNS, tPA is a well-known serine protease expressed and released in the extracellular space by many cell types including neurons.^11, 12, 13, 14^ Among the reported receptors of tPA in the CNS, one is NMDAR with GluN1 as a possible binding site.^22^ In this study, we provide molecular evidence that tPA is a ligand of NMDAR. We first demonstrate that tPA directly interacts with the GluN1 NTD of NMDARs and identify the lysine in position 178 in the GluN1 NTD as its binding site. These data are in agreement with previous demonstrations that the tPA-induced potentiation of NMDAR signaling involves the LBS contained in the K2.^33, 34^ Thus, we can postulate that this interaction of tPA with the lysine 178 of the GluN1 NTD is the first and necessary step of a previously suggested two-step process, which also involves arginine in position 260 of the GluN1 NTD.^36^ To date, the cleavage of the amino-terminal domain of the GluN1 subunit by tPA is still debated. However, there is no doubt about the capacity of tPA to enhance the NMDAR signaling.^24^ Further investigation is thus needed to determine whether GluN1 cleavage is necessary for the enhancement of NMDAR function by tPA or whether GluN1 cleavage is the result of a bystander effect, inhibits desensitization of NMDA receptors or has other functions. Also interesting, plasmin (which is generated by a tPA-dependent processing of the plasminogen) has also been reported to cleave NMDARs, specifically the GluN2 subunit. This cleavage can occur at two sites: lysine 317 on GluN2A, which relieves Zn^2+^ inhibition and thereby increases NMDAR function,^37^ and arginine 67 on GluN2B, which increases sensitivity of the NMDA receptor to glycine.^38^

NMDARs are diverse in their molecular subunit composition, their pharmacological properties and their subcellular localization. The dynamics and pharmacological features of NMDAR NTDs are critical for the control of the functional and pharmacological diversities of NMDARs. Although the regulatory functions of the GluN2 NTDs are well documented,^9^ the functions of the GluN1 NTD remain largely unknown. In a recent study, it was showed that GluN1 NTD was highly mobile and actively participated in defining the gating and pharmacological profile of NMDAR. As proposed by Paoletti's group, this discovery redefines the possible functional consequences of interactions between GluN1 NTDs of NMDAR and extracellular molecules.^10^ Severals studies propose that ifenprodil and polyamines are involved in the modification of NMDAR conformation by shielding negative charges present on GluN1 and GluN2B NTD lower lobes.^39, 40, 41^ In this study, we propose that tPA is one of these extracellular molecules capable of binding to the GluN1 NTD and, as such, to influence NMDAR signaling. Accordingly, antibodies that target the binding site of such molecules, like Glunomab, are putative modulators of NMDARs. Surface trafficking has recently emerged as a crucial cellular pathway involved in the pathophysiological tuning of excitatory synapse transmission.^42, 43^ Through the use of single particle imaging in neurons, we showed that, by selectively controlling extrasynaptic NMDARs diffusion, tPA has a key role in synaptic adaptation processes and functions of NMDARs signaling. It is important to note that tPA did not influence the percentages of detected synaptic and extrasynaptic NMDARs, respectively.

Although the relationship between the surface dynamics of NMDAR and their signaling has yet to be proven, our present data support a strong relationship between surface dynamics and subsequent signaling. By increasing the surface dynamics of a fraction of extrasynaptic NMDAR, we can propose that cumulative effects in the whole population of NMDARs are responsible for the potentiating effect of tPA on NMDARs signaling. These observations fit with our previous finding that tPA-promoted neurotoxicity through a mechanism that involved extrasynaptic and possibly GluN2D-containing NMDARs,^35, 44^ a phenomenon also associated with an activation of the ERK(½) pathway.^35^ In this study, we show that Glunomab is also capable to prevent the tPA-dependent increase of neuronal ERK(½) activation. Moreover, the neurotoxicity of tPA can be explained by the conformation (single or two chain tPA; sc-tPA or tc-tPA) and by its concentration.^45^ sc-tPA (conformation using in this study) decreased NMDAR-dependent calcium influx at 10 nM causing a neuroprotective effect, 300 nM tPA led to an increased NMDAR signaling and neurotoxicity. This protective effect of low doses of tPA was also reported by the group of Manuel Yepes.^46^ Accordingly, although tPA promotes NMDA-induced excitotoxicity,^22^ Glunomab appears to be a promising neuroprotective tool. These data are also in agreement with the proposal that the activation of synaptic NMDARs promotes cell survival, whereas activation of extrasynaptic NMDARs promotes cell death.^2^ Interestingly, a non-proteolytic form of tPA failed to promote the surface dynamics of NMDAR, suggesting that at least the catalytic site of tPA is required for this process. Whether the cleavage of GluN1 is needed,^22^ remains to be investigated. Although the use of general NMDARs blockers for the treatment of disorders of the CNS is associated with unacceptable effects,^47^ the present data suggest that targeting the potentiating effect of tPA on extrasynaptic NMDARs, may offer promising therapeutic avenues.

In conclusion, we identified the extracellular serine protease tPA, produced and released by neurons,^11, 12, 23^ as a ligand of the GluN1 NTD leading to a specific increase of the surface dynamics of extrasynaptic NMDARs, their synaptic diffusion and subsequent neurotoxicity.

## Materials and Methods

### Ethical statement

Experiments were performed in accordance with French ethical laws (act no. 87–848; Ministère de l'Agriculture et de la Forêt) and European Communities Council Directives of 24 November 1986 (86/609/EEC) guidelines for the care and use of laboratory animals, and were approved by the local and regional ethics committees (authorization code CENOMEXA 0113-03). All efforts were made to limit animal suffering. None of the experimental procedures induced animal mortality except one mouse during surgery (Figure 8 condition NMDA+tPA i.v.). All experiments were performed following the ARRIVE guidelines (www.nc3rs.org.uk), including randomization for the administration of test substances as well as analyses that were performed in a blinded manner. Male mice were housed on a 12-h light (0700 hours)/dark (1900 hours) cycle with *ad libitum* access to water and rodent chow (10 mice per cage).

### Chemicals

(5 *R*,10 *S*)-(−)-5-Methyl-10,11-dihydro-5*H*-dibenzo[*a*,*d*]cyclohepten-5,10-iminemaleate (MK801) and *N*-methyl-d-aspartate (NMDA), 2-amino-5-phosphonovaleric acid (AP5) were purchased from Tocris (Bristol, UK). Human tPA (Actilyse) was from Boehringer Ingelheim (Paris, France). tPA buffer was 34.84 mg/ml arginine, 10.72 mg/ml phosphoric acid and 0.1 mg/ml Tween 80. 5-Bromo-4-chloro-3-indolyl phosphate, alkaline-phosphatase-conjugated goat anti-mouse IgG, 3-(4,5-dimethylthiazol-2-yl)-2,5 diphenyltetrazolium bromide, lipofectamine 2000, Dulbecco's modified Eagle's medium (DMEM), poly-d-lysine, laminin, glutamine, cytosine β-d-arabinoside (Ara-C), glycine, HEPES, phosphate-buffered saline (PBS) solution, Freund adjuvants, Trypan blue solution (0.4%), paraformaldehyde, anti-mouse, anti-goat/sheep peroxidase antibody, anti-β-actin (A2066) were purchased from Sigma-Aldrich (L'Isle d'Abeau, France). Goat anti-rabbit IgG and anti-rmouse IgG were purchased from Thermofisher (Villebon-sur-Yvette, France). FuGENE-6 was purchased from Promega(Charbonnières-les-Bains, France). TRITC-, FITC-, Dye Light 649 nm-conjugated secondary antibodies were purchased from Jackson ImmunoResearch Laboratories(West Grove, PA, USA). Polyclonal antibodies raised against C-terminal end of GluN1 (Cter-GluN1) subunit was purchased from Santa Cruz (sc 1467, Heidelberg, Germany). Anti-NeuN antibody was purchased from Abcam (ab131624, Paris, France). Protein A sepharose was purchased from GE Healthcare (Orsay, France). ECL-Plus detection system was purchased from PerkinElmer Life and Analytical Sciences (Cambridge, UK). Polyclonal antibodies raised against GluN1 NTD subunit was purchased from Alomone Labs (AGC 001, Jerusalem, Israel). QDs 655 labeled goat F(ab')_2_ anti-mouse (Q11021) and anti-rabbit IgG (Q11421), secondary anti-mouse Alexa 568 antibody (A11004), Green MitoTracker (M7514), RPMI 1640 medium, Fura-2 AM, fetal bovine serum and horse serum, neurobasal medium, B27 supplement (50X) were purchased from Life Technologies (Saint Aubin, France). Lactate deshydrogenase (LDH) detection kit and mouse isotyping test kit were purchased from Roche Diagnostics (Mannheim, Germany). The anti-phosphorylated Erk(½) and anti-Erk(½) antibodies were purchased from Cell Signaling (Saint Quentin Yvelines, France).

### Single particle (QD) tracking and immunochemistry

Hippocampal neurons from E18 Sprague–Dawley rats were cultured as previously described.^48^ Cells were plated on poly-lysine pre-coated cover slips and maintained in a 3% horse serum containing neurobasal medium. After 3 day *in vitro* (DIV), the original medium was replaced with a serum-free medium. Cultures were maintained at 37 °C in 5% CO_2_. To investigate the impact of tPA on NMDAR diffusion, cultures were pre-incubated 45 min with either active tPA (300 nM, 37 °C) or tPAm (non-proteolytic tPA, 300 nM, 37 °C). QD detection and subsequent analysis were performed as previously described.^7^ Briefly, hippocampal neurons (11–12 DIV) were incubated for 10 min (37 °C) with polyclonal antibodies against GluN1 NTD subunit (Alomone Labs; 1 : 200). Neurons were then washed and incubated for 10 min (37 °C) with QDs 655 goat F(ab')_2_ anti-rabbit (1 : 10 000). Green MitoTracker (1 : 1000) was used as a synaptic marker. For the data illustrated in Figure 7, neurons were incubated for 10 min (37 °C) with either the control anti-GluN1 NTD subunit (1 : 200) or the Glunomab antibodies (1 : 200). The same protocol was followed, except that neurons were incubated with QD 655 goat F(ab')_2_ anti-rabbit or anti-mouse. Signals were detected using a EM-CCD camera (Quantem, Roper Scientific, Evry, France). QDs were followed on randomly selected dendritic regions for up to 20 min. QD recording sessions were processed with the Metamorph software (Universal Imaging Corporation, Chester, PA, USA). Instantaneous diffusion coefficient distributions of GluN1-QD are represented using median and interquartile range. The top white portion, the line between the white and the black portions and the bottom black portion correspond of the first quartile, the median and the third quartile, respectively.

### Calcium video imaging and x-link protocol in neurons

Cortical neurons were prepared from E14.5 Swiss mice as previously described.^35^ Cortices were dissected and dissociated in DMEM, and plated on 35 mm dishes previously coated with poly-lysine (0.1 mg/ml) and laminin (0.02 mg/ml). Cells were cultured in DMEM supplemented with 5% fetal bovine serum, 5% horse serum and 2 mM glutamine. Cultures were maintained at 37 °C in 5% CO_2_. Cultures were transferred into a serum-free HEPES-buffered saline solution (HBBSS: NaCl 116 mM, KCl 5.4 mM, CaCl_2_ 1.8 mM, MgSO_4_ 0.8 mM, HEPES 12 mM, NaH_2_PO_4_ 0.34 mM, d-glucose 5.5 mM, NaHCO_3_ 25 mM, Glycine 10 *μ*M) at DIV 12 and loaded with 10 *μ*M Fura-2 AM for 45 min at 37 °C. The Ca^2+^ bound form of Fura-2 gets excited at 340 nm, the Ca^2+^ unbound form at 380 nm and both recorded at 510 nm. Neurons were washed and NMDA stimulations (2 × 25–50 *μ*M for 30 s) were applied using a peristaltic pump. Neurons were then treated with monoclonal antibodies named Glunomab (10 *μ*g/ml), and/or, its isotypic control 6C9B6 (10* μ*g/ml), and/or tPA (300 nM) (Figures 2 and 4,Supplementary Figure 1) and/or plasmin (100 *μ*g/ml) (Figure 2) during 45 min. For each cell, the area under curve (AUC) corresponding to the intracellular calcium influx induced by NMDA (ratio 340/380) was calculated before (AUCb) (mean of the two first pre-treatment stimulations) and after treatment (AUCa) (tPA, plasmin, Glunomab, 6C9B6, x-link) (mean of the 2 s post-treatment stimulations). We then compared, for each cell, the amount of NMDA-induced calcium influx (AUC) after treatment with the NMDA-induced calcium influx recorded before treatment. Thus, each cell is its own control with an expression of the modification of the NMDA-induced calcium influx because of the treatment performed between the two rounds of stimulations, expressed as a percentage of response to treatments (% of responsiveness).


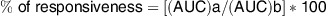


with (AUC)a: sum of area under curve of Fura-2 ratio during both NMDA stimulations after treatment and (AUC)b: sum of area under curve of Fura-2 ratio during NMDA stimulations before treatment.

Experiments were performed at room temperature, on the stage of a Leica DMI6000B inverted microscope equipped with a 150 W Xenon high stability lamp and a Leica 40 × , 1.3 numerical aperture epifluorescence oil immersion objective (Wetzlar, Germany). Fura-2 (excitation: 340, 380 nm, emission: 510 nm) ratio images were acquired with a Digital CMOS camera (Hamamatsu, ORCA-Flash2.8 C11440-10C, Massy, France) and digitized (2048 × 2048 pixels) using Metafluor 6.1 software (Universal Imaging Corporation). For x-link procedures, neurons were treated for 15 min with primary anti-GluN1 (1 : 20) and secondary (1 : 30) antibodies (x-link) (Figure 3 and Supplementary Figure 1). The buffer of Glunomab, control 6C9B6 and x-link is PBS solution.

### Production and purification of monoclonal antibodies and ELISA

Eight-week-old Balb/C male mice (20–25 g) were immunized by intraperitoneal injection of immunogenic mixtures: complete Freund adjuvant (first injection) and incomplete Freund adjuvant (once a week during 3 weeks) alone (control) or containing the rHis-GluN1 NTD. Sp2/0Ag14 myeloma cell line (ATCC, CRL 1581, Molsheim, France) was fused with splenocytes from selected immunized mouse according to standard protocols and fusion product was plated in culture microplates for 15 days before primary screening.

Clone selection was performed using indirect ELISA along this process: from primary screening, confirmation to sub-cloning screenings. Antigens used were rHis-GluN1 NTD and His-mock as negative control. After primary screening and confirmation, two antibody-producing hybridomas were identified and were sub-cloned twice then frozen in liquid nitrogen. Monoclonal antibodies were produced *in vitro* after collecting concentrated supernatants. Purifications were done by affinity chromatography on protein A sepharose. The mAbs were isotyped with a mouse isotyping test kit according to the manufacturer's recommendations.

6C9B6 is a clone produced in parallel to Glunomab. Like Glunomab, it binds recombinant NTD of GluN1, but it does not block the capacity of tPA to bind to and promote NMDAR signaling, thus targeting a different epitope than GluN1.

### Immunoblotting and immunoprecipitation

Proteins (20 *μ*g) were resolved on 7.5% SDS-PAGE blots under denaturing conditions and transferred onto a polyvinylidene difluoride membrane. Membranes were blocked with Tris-buffered saline (10 mM Tris and 200 mM NaCl, pH 7.4) containing 0.05% Tween 20 and 0.4% BSA. Blots were incubated overnight with primary antibodies: Glunomab (clone 15A4B2, 0.5 mg/ml, 1 : 1000) or Cter-GluN1 antibody (1 : 250). After incubation with the anti-mouse or anti-goat peroxidase-conjugated secondary antibody, proteins were visualized with an enhanced chemiluminescence ECL-Plus detection system. For the immunoprecipitation, supernatants from TNT buffer (50 mM Tris-HCl, pH 7.4, 150 mM NaCl, and 0.5% Triton 100X)-lysed tissues or cultures (500 *μ*g of proteins) were incubated overnight at 4 °C in the presence of the Cter-GluN1 antibody (2 *μ*g). Samples were then coupled to protein G-sepharose beads as described by the manufacturer (GE Healthcare, Orsay, France). Proteins were separated by 7.5% SDS-PAGE, blots were exposed in the presence of Glunomab (1 : 1000) and revealed following the procedure of immunoblotting described above.

### Immunohistochemistry

Adults mice (Swiss males; 35–45 g) and rats (Sprague–Dawley males, 350–400 g) were deeply anesthetized and perfused transcardially with 4% paraformaldehyde in 150 ml or 400 ml of 0.1M sodium phosphate buffer, respectively. Coronal sections of brain and liver (10 *μ*m) were incubated overnight at room temperature with Glunomab (1 : 500), anti-Cter-GluN1 antibody (1 : 400) and anti-NeuN antibody (1 : 250). Detection was performed using F(ab')2 fragments of donkey anti-goat linked TRITC, anti-mouse linked FITC or anti-chicken linked Dylight 649 nm (1 :  600). All sections were examined with a Leica DM6000 microscope. Images were captured using a CoolSnap camera and visualized with Metavue 5.0 software (Molecular Devices, Sunnyvale, CA, USA).

### Epitope mapping and Alascan

Epitope mapping and Alascan are presented in Supplementary Figure 3. A total of 141 overlapping pentadecapeptides, frame-shifted by three or one residue covering the entire amino-acid sequence of GluN1 NTD (residues 19–371), were prepared using the Spot technique according to the protocol previously described by Laune *et al.*^49^ Briefly, peptides were assembled using Fmoc chemistry on a cellulose membrane containing an aminopolyethyleneglycol moiety. The C-terminal residue of each peptide was coupled to the moiety. After Fmoc deprotection, the other amino acids were sequentially added as in conventional solid-phase peptide synthesis. Finally, the side-chain protecting groups were removed by trifluoroacetic acid treatment in the presence of appropriate scavengers, whereas the linkage of the peptides to the membrane was maintained. Free cysteines were replaced with non-reactive acetamidomethyl cysteines. Once reactive peptides were identified, alanine scanning of positive sequences was performed to identify key residues contributing to the epitope; signal extinction indicated a key contribution of the aa whereas a decrease in the signal meant partial contribution to the epitope.

Purified mAbs were incubated on the membrane, and then antibody binding was detected by using an alkaline-phosphatase-conjugated goat anti-mouse IgG. For staining, 5-bromo-4-chloro-3-indolyl phosphate (BCIP) and 3-(4,5-dimethylthiazol-2-yl)-2,5 diphenyltetrazolium bromide (MTT) were used as substrates. A blue precipitate was observed on peptides to which the antibody was bound. To allow the re-use of the membrane, it was sequentially treated with dimethylformamide, then 1% SDS, 0.1% 2-mercaptoethanol in 8 M urea, then ethanol/water/acetic acid (50 : 40 : 10 vol/vol/vol) and, finally, ethanol in order to remove the precipitated dye and molecules bound to the peptides.

### Transfection and calcium video imaging in HEK-293 cells

Human embryonic kidney 293 cells (HEK-293 cells) were grown in RPMI 1640 supplemented with 5% fetal bovine serum and with NMDA antagonists (200 *μ*M AP5 and 2 mM MgCl_2_). Cells were transfected by lipofection (8*μ*l, FuGENE-6), with a mixture containing 2 *μ*g of GluN1-1b WT (or GluN1-1b K178V or GluN1-1b K190V; Figure 5) and 2 *μ*g of GluN2A or with tPA WT or tPA K2* (Figure 6). Transfected HEK-293 cells were loaded with 10 *μ*M fura-2 AM for 30 min at 37 °C in HBSS containing 0.1% pluronic F-127, 20% solution in DMSO, AP5 (200 *μ*M) and MgCl_2_ (2 mM) and then incubated for an additional 45-min period in HBBSS prior recording. We used the same set up and data analysis as mentioned in the methods section for calcium video imaging in neurons. Concentrations of tPA and Glunomab were also of 300 nM and 10 *μ*g/ml, respectively. NMDA exposures were performed at 100 *μ*M for 30 s and the duration of treatments was of 20 min between the two rounds of NMDA stimulations.

### Site-directed mutagenesis

Mutagenesis of rat wt-GluN1-1b (GluN1-1b K178V or GluN1-1b K190V) was performed with the QuikChange XL Kit (VWR International France, Fontenay-sous-Bois, France) and the following primers: 5′-GGCAGCGCAGgtGCGCTTGGAG-3′ and 5′-CGTCCCTCGTGGTCGTCG-3′ (to GluN1-1b K178V), 5′-ACGGGAGTCCgtgAGTAAAAAAAGGAACTATG-3′ and 5′-TCCTCCAGCAACGTCTCC-3′ (to GluN1-1b K190V). Mutations were confirmed by sequence analysis. Site-directed mutagenesis strategy in K2 domain of tPA sequencing procedure have been described previously.^33^

### Excitotoxic neuronal death

Cultures of cortical neurons at 12–13 DIV were prepared in same way as that reported above for calcium video imaging. Neurons were rinsed three times with serum-free medium and as previously described,^35^ excitotoxicity was induced by exposure to NMDA (12.5 *μ*M) for 24 h. NMDA was applied alone or together with tPA (300 nM) and/or Glunomab (0.1, 1 or 10 *μ*g/ml). Twenty-four hours later, neuronal death was quantified by measurement of LDH released from damaged cells.

### *In vivo* excitotoxic lesions

Twelve-week-old Swiss mice (male) were anesthetized (2% of isoflurane in 1/3 O_2_ and 2/3 N_2_O) and placed in a stereotaxic frame prior injection. A cortical unilateral injection (coordinates: 0.5 mm posterior, 3.0 mm lateral, –0.8 mm ventral to the bregma; stereotaxic atlas G Paxinos & KBJ Franklin) of NMDA (10 nmoles, 1 *μ*l) was performed. Fifteen minutes after the excitotoxic lesion, a 200 *μ*l intravenous bolus injection of tPA (10 mg/kg) or saline with or without Glunomab (160 *μ*g) was given via a catheter previously inserted into the tail vein. Solutions were injected by the use of a micropipette made with hematologic micropipettes. Twenty-four hours later, mice were killed and the brains removed and frozen in isopentane. Cryostat-cut coronal brain sections (20 *μ*m) were stained with thionine and a volumetric analysis of brain lesions was performed with the Image J software (NIH software, National Institute of Health, Bethesda, MD, USA). Region of interest were determined through the use of a stereotoxic atlas for the mouse and an image analysis system (Scion Image Scion Corporation, Frederick, MD, USA) was used to measure lesion corresponding to the nonstained area (*n*=7, 8 or 9 per group).

### P-Erk(½)-dependent NMDAR signaling

Erk(½) activation by phosphorylation was used as an index of NMDAR signaling. NMDARs were activated by exposure of the primary neuronal cultures to NMDA (50 *μ*M), tPA (300 nM) and Glunomab (10 *μ*g/ml) either alone or in combination. After 5 min of treatment, cells were chilled on ice and lysed in buffer containing Tris-NaCl-Triton 1% of protease inhibitor cocktail and 1% of phosphatase inhibitor cocktail. Lysates were clarified by centrifugation at 13 000 *g*, for 10 min at 4 °C. Proteins were quantified and immunoblotted using adequate primary antibodies (anti-phosphorylated Erk(½) and anti-Erk(½), 1 : 1000) followed by incubation with the appropriate peroxydase-conjugated secondary antibody. Erk(½) and p-Erk(½) levels were investigated by running separate gels from the same protein extracts.

### Statistical analysis

Normality was tested for all analyses of variance. The instantaneous diffusion coefficients (Figures 1d and 7e) is reported as the median±25–75% (interquartile range, IQR). The other data of Figures 1 and 7 are expressed as mean±S.E.M. Comparisons between groups for instantaneous diffusion coefficient were performed with Kruskal–Wallis test followed by Dunn's multiple comparison test as post-hoc test. Comparison between extrasynaptic fractions (Figure 1e) was performed with one-way ANOVA. Significance levels were defined as *P<0.05, **P<0.01, ***P<0.001 and not significant (NS). For calcium video imaging of neurons (Figures 2,3 and 4 and Supplementary Figure 1) and of HEK-293 cells (Figures 5 and 6), the responsiveness was analyzed by Wilcoxon signed-rank test to compare pre-and post-incubation NMDA responses. Significance levels were defined as ^#^*P*<0.0001 (for neurons experiments) and ^#^*P*<0.01 (for HEK-293 cells experiments). In addition, for group comparison, Kruskal–Wallis tests were used followed by Mann–Whitney *U*-tests as post-hoc tests, significance levels was defined as *P<0.05, **P<0.01, ***P<0.001, ****P<0.0001 and NS. Results are expressed as mean±S.E.M. For Elisa (Figure 4), the excitotoxic neuronal death (Figures 8a and b), *in vivo* excitotoxic lesions (Figure 8d), P-Erk(½)-dependent NMDAR signaling (supplementary Figure 4), Kruskal–Wallis tests were used followed by Mann–Whitney *U*-test as post-hoc test. Results are expressed as mean±S.E.M., significance levels was defined as *P<0.05 and NS.

## Abbreviations

Cter GluN1: C-terminal end of GluN1 subunit
DIV: day *in vitro*
GluN: NMDA receptor subunit
K2: kringle 2 domain of tPA
LBS: lysine-binding site
NMDAR: *N*-methyl-d-aspartate receptor
NTD: N-terminal domain
QD: quantum dot
TMD: transmembrane domain
tPA: tissue-type plasminogen activator
WT: wild-type.

## References

[bib1] Paoletti P, Bellone C, Zhou Q. NMDA receptor subunit diversity: impact on receptor properties, synaptic plasticity and disease. Nat Rev Neurosci 2013; 14: 383–400. 23686171 10.1038/nrn3504

[bib2] Hardingham GE, Bading H. Synaptic versus extrasynaptic NMDA receptor signalling: implications for neurodegenerative disorders. Nat Rev Neurosci 2010; 11: 682–696. 20842175 10.1038/nrn2911 PMC2948541

[bib3] Groc L, Heine M, Cousins SL, Stephenson FA, Lounis B, Cognet L et al. NMDA receptor surface mobility depends on NR2A-2B subunits. Proc Natl Acad Sci USA 2006; 103: 18769–18774. 17124177 10.1073/pnas.0605238103 PMC1693737

[bib4] Bard L, Sainlos M, Bouchet D, Cousins S, Mikasova L, Breillat C et al. Dynamic and specific interaction between synaptic NR2-NMDA receptor and PDZ proteins. Proc Natl Acad Sci USA 2010; 107: 19561–19566. 20974938 10.1073/pnas.1002690107 PMC2984211

[bib5] Triller A, Choquet D. Surface trafficking of receptors between synaptic and extrasynaptic membranes: and yet they do move!. Trends Neurosci 2005; 28: 133–139. 15749166 10.1016/j.tins.2005.01.001

[bib6] Michaluk P, Mikasova L, Groc L, Frischknecht R, Choquet D, Kaczmarek L. Matrix metalloproteinase-9 controls NMDA receptor surface diffusion through integrin beta1 signaling. J Neurosci 2009; 29: 6007–6012. 19420267 10.1523/JNEUROSCI.5346-08.2009 PMC6665240

[bib7] Groc L, Lafourcade M, Heine M, Renner M, Racine V, Sibarita JB et al. Surface trafficking of neurotransmitter receptor: comparison between single-molecule/quantum dot strategies. J Neurosci 2007; 27: 12433–12437. 18003820 10.1523/JNEUROSCI.3349-07.2007 PMC6673310

[bib8] Papouin T, Ladépêche L, Ruel J, Sacchi S, Labasque M, Hanini M et al. Synaptic and extrasynaptic NMDA receptors are gated by different endogenous coagonists. Cell 2012; 150: 633–646. 22863013 10.1016/j.cell.2012.06.029

[bib9] Cull-Candy SG, Leszkiewicz DN. Role of distinct NMDA receptor subtypes at central synapses. Sci STKE 2004; re16. 10.1126/stke.2552004re16 15494561

[bib10] Zhu S, Stroebel D, Yao CA, Taly A, Paoletti P. Allosteric signaling and dynamics of the clamshell-like NMDA receptor GluN1 N-terminal domain. Nat Struct Mol Biol 2013; 20: 477–485. 23454977 10.1038/nsmb.2522

[bib11] Shin CY, Kundel M, Wells DG. Rapid, activity-induced increase in tissue plasminogen activator is mediated by metabotropic glutamate receptor-dependent mRNA translation. J Neurosci 2004; 24: 9425–9433. 15496678 10.1523/JNEUROSCI.2457-04.2004 PMC6730095

[bib12] Siao CJ, Fernandez SR, Tsirka SE. Cell type-specific roles for tissue plasminogen activator released by neurons or microglia after excitotoxic injury. J Neurosci 2003; 23: 3234–3242. 12716930 10.1523/JNEUROSCI.23-08-03234.2003 PMC6742309

[bib13] Lemarchant S, Pruvost M, Hébert M, Gauberti M, Hommet Y, Briens A et al. tPA promotes ADAMTS-4-induced CSPG degradation, thereby enhancing neuroplasticity following spinal cord injury. Neurobiol Dis 2014; 66C: 28–42. 10.1016/j.nbd.2014.02.005 24576594

[bib14] Bukhari N, Torres L, Robinson JK, Tsirka SE. Axonal regrowth after spinal cord injury via chondroitinase and the tissue plasminogen activator (tPA)/plasmin system. J Neurosci 2011; 31: 14931–14943. 22016526 10.1523/JNEUROSCI.3339-11.2011 PMC3206287

[bib15] Yepes M, Roussel BD, Ali C, Vivien D. Tissue-type plasminogen activator in the ischemic brain: more than a thrombolytic. Trends Neurosci 2009; 32: 48–55. 18963068 10.1016/j.tins.2008.09.006

[bib16] Polavarapu R, Gongora MCYi H, Ranganthan S, Lawrence DAStrickland D et al. Tissue-type plasminogen activator-mediated shedding of astrocytic low-density lipoprotein receptor-related protein increases the permeability of the neurovascular unit. Blood 2007; 109: 3270–3278. 17170123 10.1182/blood-2006-08-043125 PMC1852247

[bib17] Samson AL, Medcalf RL. Tissue-type plasminogen activator: a multifaceted modulator of neurotransmission and synaptic plasticity. Neuron 2006; 50: 673–678. 16731507 10.1016/j.neuron.2006.04.013

[bib18] Madani R, Hulo S, Toni N, Madani H, Steimer T, Muller D et al. Enhanced hippocampal long-term potentiation and learning by increased neuronal expression of tissue-type plasminogen activator in transgenic mice. EMBO J 1999; 18: 3007–3012. 10357813 10.1093/emboj/18.11.3007 PMC1171382

[bib19] Pawlak R, Nagai N, Urano T, Napiorkowska-Pawlak D, Ihara H, Takada Y et al. Rapid, specific and active site-catalyzed effect of tissue-plasminogen activator on hippocampus-dependent learning in mice. Neuroscience 2002; 113: 995–1001. 12182903 10.1016/s0306-4522(02)00166-5

[bib20] Pawlak R, Magarinos AM, Melchor J, McEwen B, Strickland S. Tissue plasminogen activator in the amygdala is critical for stress-induced anxiety-like behavior. Nat Neurosci 2003; 6: 168–174. 12524546 10.1038/nn998

[bib21] Liot G, Roussel BD, Lebeurrier N, Benchenane K, López-Atalaya JP, Vivien D et al. Tissue-type plasminogen activator rescues neurones from serum deprivation-induced apoptosis through a mechanism independent of its proteolytic activity. J Neurochem 2006; 98: 1458–1464. 16800849 10.1111/j.1471-4159.2006.03982.x

[bib22] Nicole O, Docagne F, Ali C, Margaill I, Carmeliet P, MacKenzie ET et al. The proteolytic activity of tissue-plasminogen activator enhances NMDA receptor-mediated signaling. Nat Med 2001; 7: 59–64. 11135617 10.1038/83358

[bib23] Wu F, Wu J, Nicholson AD, Echeverry R, Haile WB, Catano M et al. Tissue-type plasminogen activator regulates the neuronal uptake of glucose in the ischemic brain. J Neurosci 2012; 32: 9848–9858. 22815500 10.1523/JNEUROSCI.1241-12.2012 PMC3437989

[bib24] Samson AL, Nevin ST, Croucher D, Niego B, Daniel PB, Weiss TW et al. Tissue-type plasminogen activator requires a co-receptor to enhance NMDA receptor function. J Neurochem 2008; 107: 1091–1101. 18796005 10.1111/j.1471-4159.2008.05687.x PMC3198853

[bib25] Groc L, Heine M, Cognet L, Brickley K, Stephenson FA, Lounis B et al. Differential activity-dependent regulation of the lateral mobilities of AMPA and NMDA receptors. Nat Neurosci 2004; 7: 695–696. 15208630 10.1038/nn1270

[bib26] Heine M, Groc L, Frischknecht R, Béïque JC, Lounis B, Rumbaugh G et al. Surface mobility of postsynaptic AMPARs tunes synaptic transmission. Science 2008; 320: 201–205. 18403705 10.1126/science.1152089 PMC2715948

[bib27] Groc L, Choquet D, Chaouloff F. The stress hormone corticosterone conditions AMPAR surface trafficking and synaptic potentiation. Nat Neurosci 2008; 11: 868–870. 18622402 10.1038/nn.2150

[bib28] Dupuis JP, Ladépêche L, Seth H, Bard L, Varela J, Mikasova L et al. Surface dynamics of GluN2B-NMDA receptors controls plasticity of maturing glutamate synapses. EMBO J 2014; 33: 842–861. 24591565 10.1002/embj.201386356 PMC4194110

[bib29] Mikasova L, De Rossi P, Bouchet D, Georges F, Rogemond V, Didelot A et al. Disrupted surface cross-talk between NMDA and Ephrin-B2 receptors in anti-NMDA encephalitis. Brain 2012; 135: 1606–1621. 22544902 10.1093/brain/aws092

[bib30] Macrez R, Obiang P, Gauberti M, Roussel B, Baron A, Parcq J et al. Antibodies preventing the interaction of tissue-type plasminogen activator with N-methyl-D-aspartate receptors reduce stroke damages and extend the therapeutic window of thrombolysis. Stroke 2011; 42: 2315–2322. 21680906 10.1161/STROKEAHA.110.606293

[bib31] Kvajo M, Albrecht H, Meins M, Hengst U, Troncoso E, Lefort S et al. Regulation of brain proteolytic activity is necessary for the *in vivo* function of NMDA receptors. J Neurosci 2004; 24: 9734–9743. 15509762 10.1523/JNEUROSCI.3306-04.2004 PMC6730139

[bib32] Benchenane K, Castel H, Boulouard M, Bluthé R, Fernandez-Monreal M, Roussel BD et al. Anti-NR1 N-terminal-domain vaccination unmasks the crucial action of tPA on NMDA-receptor-mediated toxicity and spatial memory. J Cell Sci 2007; 120: 578–585. 17244650 10.1242/jcs.03354

[bib33] Parcq J, Bertrand T, Baron AF, Hommet Y, Anglès-Cano E, Vivien D et al. Molecular requirements for safer generation of thrombolytics by bioengineering the tissue-type plasminogen activator A chain. J Thromb Haemost 2013; 11: 539–546. 23301636 10.1111/jth.12128

[bib34] López-Atalaya JP, Roussel BD, Ali C, Maubert E, Petersen KU, Berezowski V et al. Recombinant Desmodus rotundus salivary plasminogen activator crosses the blood-brain barrier through a low-density lipoprotein receptor-related protein-dependent mechanism without exerting neurotoxic effects. Stroke 2007; 38: 1036–1043. 17325305 10.1161/01.STR.0000258100.04923.84

[bib35] Baron A, Montagne A, Cassé F, Launay S, Maubert E, Ali C et al. NR2D-containing NMDA receptors mediate tissue plasminogen activator-promoted neuronal excitotoxicity. Cell Death Differ 2010; 17: 860–871. 19911010 10.1038/cdd.2009.172

[bib36] Fernández-Monreal M, López-Atalaya JP, Benchenane K, Cacquevel M, Dulin F, Le Caer JP et al. Arginine 260 of the amino-terminal domain of NR1 subunit is critical for tissue-type plasminogen activator-mediated enhancement of N-methyl-D-aspartate receptor signaling. J Biol Chem 2004; 279: 50850–50856. 15448144 10.1074/jbc.M407069200

[bib37] Yuan H, Vance KM, Junge CE, Geballe MT, Snyder JP, Hepler JR et al. The serine protease plasmin cleaves the amino-terminal domain of the NR2A subunit to relieve Zinc inhibition of the *N*-methyl-d-aspartate receptors. J Biol Chem 2009; 284: 12862–12873. 19240037 10.1074/jbc.M805123200 PMC2676017

[bib38] Ng KS, Leung HW, Wong PT, Low CM. Cleavage of the NR2B subunit amino terminus of *N*-methyl-d-aspartate (NMDA) receptor by tissue plasminogen activator: identification of the cleavage site and characterization of ifenprodil and glycine affinities on truncated NMDA receptor. J Biol Chem 2012; 287: 25520–25529. 22610100 10.1074/jbc.M112.374397 PMC3408171

[bib39] Mony L, Zhu S, Carvalho S, Paoletti P. Molecular basis of positive allosteric modulation of GluN2B NMDA receptors by polyamines. EMBO J 2011; 30: 3134–3146. 21685875 10.1038/emboj.2011.203 PMC3160180

[bib40] Tomitori H, Suganami A, Saiki R, Mizuno S, Yoshizawa Y, Masuko T et al. Structural changes of regulatory domain heterodimer of N-methyl-D-aspartate receptor subunits GluN1 and GluN2B through the binding of spermine and ifenprodil. J Pharmacol Exp Ther 2012; 343: 82–90. 22743575 10.1124/jpet.112.192286 PMC3464035

[bib41] Zhu S, Paoletti P. Allosteric modulators of NMDA receptors: multiple sites and mechanisms. Curr Opin Pharmacol 2015; 20: 14–23. 25462287 10.1016/j.coph.2014.10.009

[bib42] Lau C G, Zukin R S. NMDA receptor trafficking in synaptic plasticity and neuropsychiatric disorders. Nat Rev Neurosci 2007; 8: 413–426. 17514195 10.1038/nrn2153

[bib43] Bard L, Groc L. Glutamate receptor dynamics and protein interaction: lessons from the NMDA receptor. Mol Cell Neurosci 2011; 48: 298–307. 21640188 10.1016/j.mcn.2011.05.009

[bib44] Obiang P, Macrez R, Jullienne A, Bertrand T, Lesept F, Ali C et al. GluN2D subunit-containing NMDA receptors control tissue plasminogen activator-mediated spatial memory. J Neurosci 2012; 32: 12726–12734. 22972996 10.1523/JNEUROSCI.6202-11.2012 PMC6703808

[bib45] Bertrand T, Lesept F, Chevilley A, Lenoir S, Aimable M, Briens A et al. Conformations of tissue plasminogen activator (tPA) orchestrate neuronal survival by a crosstalk between EGFR and NMDAR. Cell Death Dis 2015; 6: e1924. 10.1038/cddis.2015.296 PMC4632317 26469972

[bib46] Wu F, Echeverry R, Wu J, An J, Haile WB, Cooper DS et al. Tissue-type plasminogen activator protects neurons from excitotoxin-induced cell death via activation of the ERK1/2-CREB-ATF3 signaling pathway. Mol Cell Neurosci 2013; 52: 9–19. 23063501 10.1016/j.mcn.2012.10.001 PMC3540185

[bib47] Parsons MP, Raymond L. Extrasynaptic NMDA receptor involvement in central nervous system disorders. Neuron 2014; 82: 279–293. 24742457 10.1016/j.neuron.2014.03.030

[bib48] Ladepeche L, Dupuis JP, Bouchet D, Doudnikoff E, Yang L, Campagne Y et al. Single-molecule imaging of the functional crosstalk between surface NMDA and dopamine D1 receptors. Proc Natl Acad Sci USA 2013; 110: 18005–18010. 24127604 10.1073/pnas.1310145110 PMC3816474

[bib49] Laune D, Molina F, Ferrières G, Villard S, Bès C, Rieunier F et al. Application of the Spot method to the identification of peptides and amino acids from the antibody paratope that contribute to antigen binding. J Immunol Methods 2002; 267: 53–70. 12135800 10.1016/s0022-1759(02)00140-0

[bib50] Farina AN, Blain KY, Maruo T, Kwiatkowski W, Choe S, Nakagawa T. Separation of domain contacts is required for heterotetrameric assembly of functional NMDA receptors. J Neurosci 2011; 31: 3565–3579. 21389213 10.1523/JNEUROSCI.6041-10.2011 PMC3063151

