# Supplementary material for: Tissue-type plasminogen activator controls neuronal death by raising surface dynamics of extrasynaptic NMDA receptors
Source: Cell Death Dis. 2016 Nov 10;7(11):e2466–. doi: 10.1038/cddis.2016.279 (PMC5260909; doi:10.1038/cddis.2016.279)
Supplement: Supplementary Information [file cddis2016279x1.pdf]

Tissue-type plasminogen activator controls neuronal death by raising surface dynamics of extrasynaptic NMDA receptors.

Flavie Lesept[1], Arnaud Chevilley*[1], Julie Jezequel*[2,3], Laurent Ladépêche[2,3], Richard Macrez[1], Margaux Aimable[1], Sophie Lenoir[1], Thomas Bertrand[1], Laëtitia Rubrecht[4], Pascale Galea[4], Laurent Lebouvier[1], Karl-Uwe Petersen[5], Yannick Hommet[1], Eric Maubert[1], Carine Ali[1], Laurent Groc[2,3], Denis Vivien[1].

<u>Summary of supplementary data:</u>

This supplementary information includes 5 figures supporting the main text, detailed data from each supplementary figure has been mentioned in the main text.

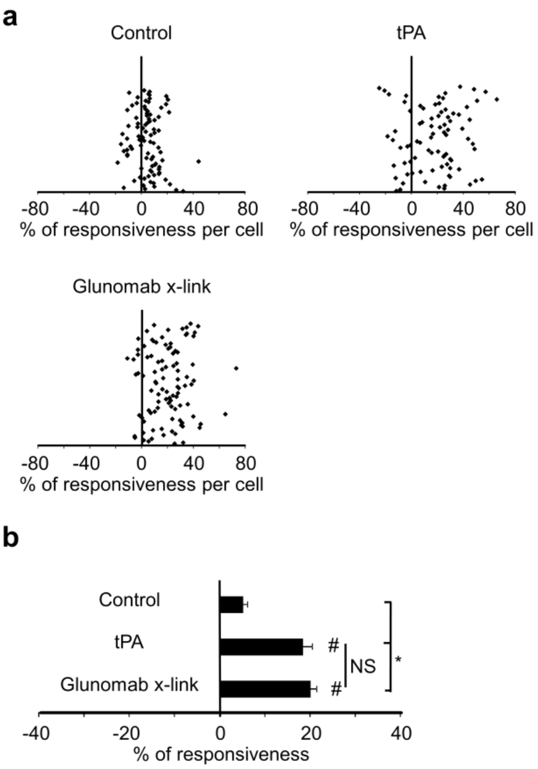

Supplementary Figure 1. Glunomab mimicks x-link-induced NMDARs clustering. (**a**) NMDA-induced calcium influx measured after the following treatments: buffer alone (n= 89 cells), tPA alone (n= 83 cells), Glunomab x-link (n= 98 cells). Each dot represents one cell. (**b**) Percentage of stimulation or inhibition after incubation were calculated for each individual cell and reported as percentages of responsiveness for each group. (mean ± SEM; N= 3 independent experiments; NS: not significant; ****: $p < 0.0001$ Kruskal-Wallis test followed by Mann-Whitney test for group comparison; #: $p < 0.0001$ Wilcoxon signed-rank test for the comparison pre- and post-incubation responses).

a

Clone Name	Supernatant dilution	His-GluN1 NTD	His-mock	ratio
15A4B2	1/2	2.8	1.6	1.7
	1/10	2.1	1.3	1.6
	1/100	0.6	0.4	1.3
	1/1000	0.2	0.1	1.4
6C9B6	1/2	2.5	1.6	1.4
	1/10	2.3	1.6	1.5
	1/100	1.9	1.3	1.5
	1/1000	1.0	0.6	1.7

b

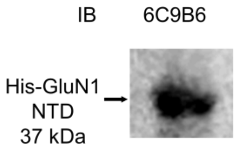

Supplementary Figure 2. Characterization and selection of positive clones. (**a**) Example of indirect ELISA assays performed with conditioned media harvested from Glunomab (clone 15A4B2) and clone 6C9B6, using a His-tagged mock protein as a control or the His-GluN1 NTD as the specific antigen. (**b**) Immunoblotting performed after SDS-PAGE resolution of recombinant His-GluN1 NTD (20 µg loaded per lane) by using the clone 6C9B6 as primary antibody. The immunoblot performed by using the clone 15A4B2 as primary antibody can be visualized figure 4c.

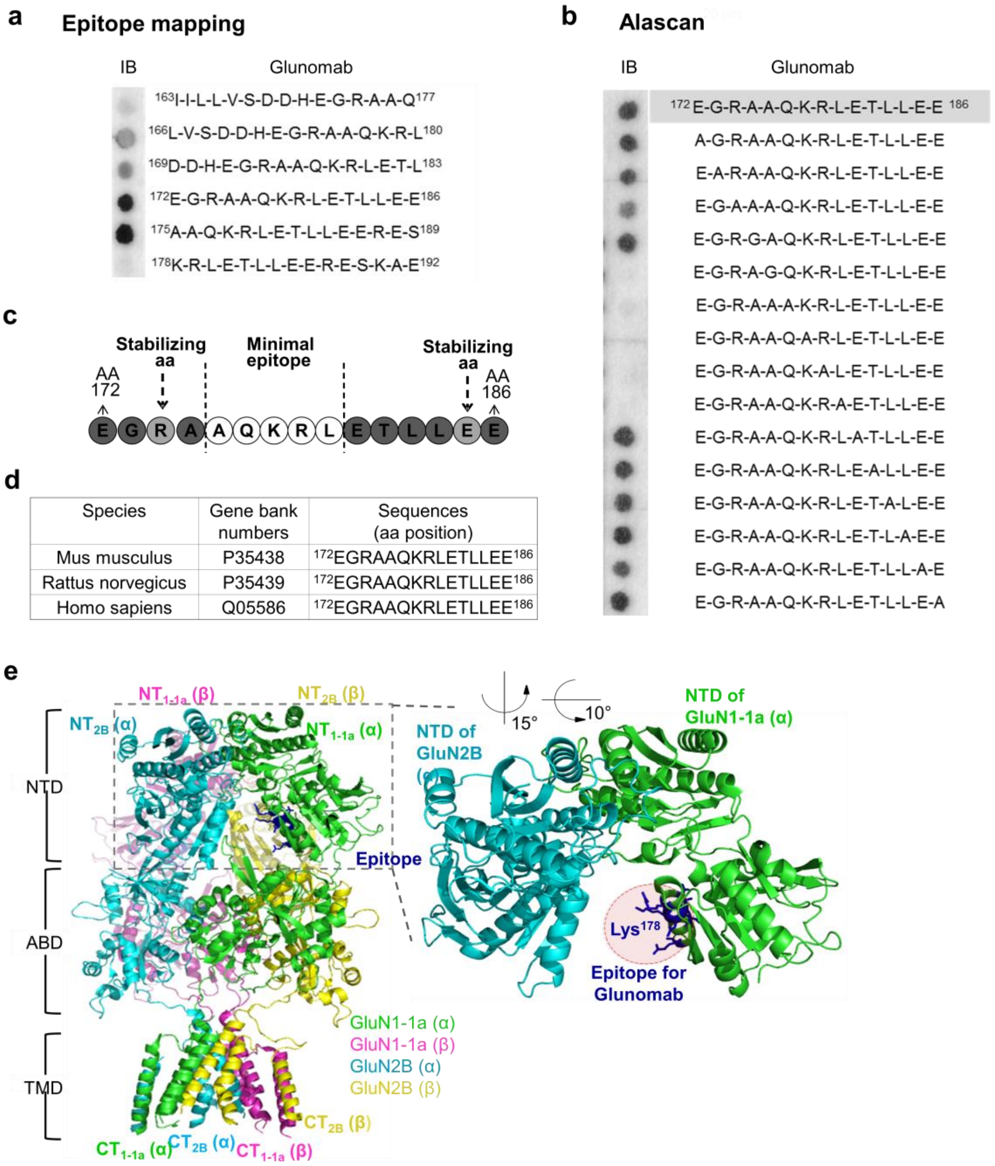

Supplementary Figure 3. Glunomab prevents tPA-mediated potentiation of NMDAR signaling. (**a**). Immunoblotting of a total of 141 overlapping pentadecapeptides frame-shifted by three residues covering a part of the amino acid sequence of GluN1 NTD (19-371aa) was revealed with Glunomab. The figure represents 6 (163-192aa) out of 141 peptides analyzed. Epitope mapping identified the putative epitope for Glunomab as being the following amino acid sequence in GluN1 NTD "^{172}EGRAAQKRLETLLEE^{186}". (**b**) Alanine scanning (Alascan) of the amino acid sequence of the GluN1 NTD "^{172}EGRAAQKRLETLLEE^{186}". (**c**) The epitope identified revealed that the key amino acids within this minimal epitope were A^{176}, Q^{177}, K^{178}, R^{179}, L^{180} and that amino acids R^{174}, E^{185} may stabilize binding of Glunomab

to GluN1 NTD.(**d**) This epitope is conserved at 100% between mice, rats and humans. (**e**) Overall structure of GluN1a-GluN2B NMDAR (PDB ID: 4PE5; ref 50). GluN1a and GluN2B subunits, labelled as GluN1-1a (α), GluN1-1a (β), GluN2B (α), GluN2B (β) are coloured in green, pink, cyan and yellow, respectively. The epitope of Glunomab is coloured in dark bleu and showed in GluN1a (α). This epitope may have possibly access to extracellular molecules which are represented by a red circle.

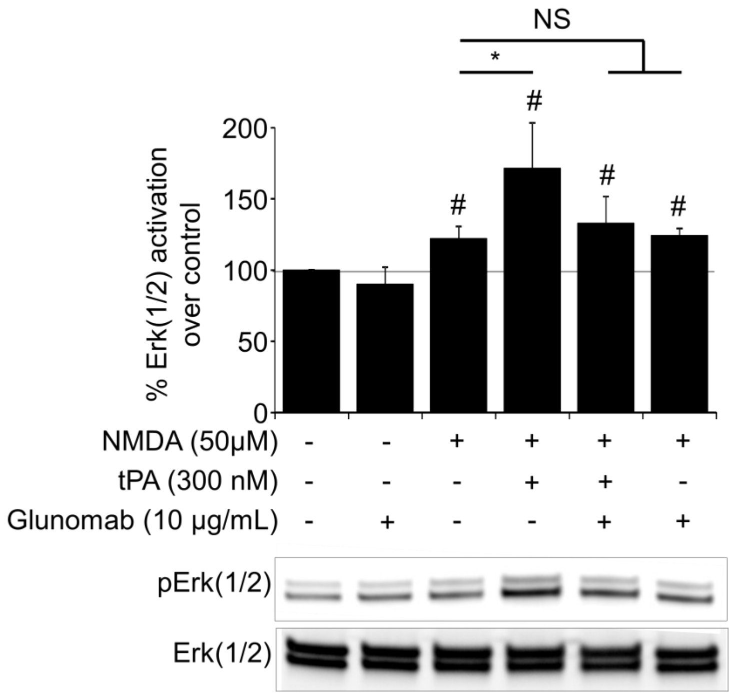

Supplementary Figure 4. Glunomab prevents the tPA-dependent increase of NMDA-mediated neuronal Erk(½) activation. Immunodetection of activated and total Erk(1/2) performed from primary cultures of cortical neurons (12–14 DIV) subjected to a 5 min exposure of NMDA either alone (50 µM) or with tPA (300nM) and/or Glunomab (10µg/mL). The graph shows the quantification of Erk(½) activation relative to control (mean ± SEM; N= 5 independent experiments, including n= 4 individual dishes per condition NS: not significant; *: $p < 0.05$; Kruskal-Wallis and Mann-Whitney tests for group comparison; #: $p < 0.05$; Kruskal-Wallis and Mann-Whitney tests for comparison with control conditions).

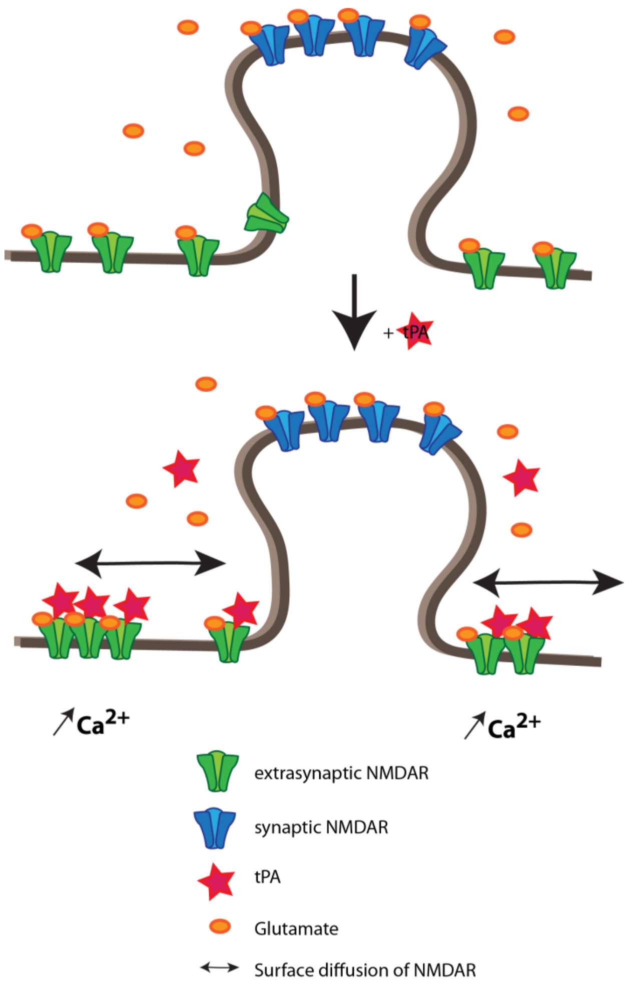

Supplementary Figure 5. tPA promotes the surface diffusion of extrasynaptic NMDAR and subsequent signaling. Interaction of tPA with extrasynaptic NMDAR promotes their capacity to diffuse at the neuronal surface, likely favouring clusters formation and subsequent NMDA-dependent calcium influx and excitotoxicity.
